# Comparison of metabolite profiles and policosanol contents in the sprout of Oriental wheat cultivars (*Triticum turgidum* ssp. *turanicum*)

**DOI:** 10.3389/fnut.2025.1649097

**Published:** 2025-09-10

**Authors:** Euna Choi, Jisu Park, Min-Jeong Hong, Chang Hyun Jin, Bo-Ram Kim, Bomi Nam, MinKyun Na, Yun-Seo Kil, Ah-Reum Han

**Affiliations:** ^1^Advanced Radiation Technology Institute, Korea Atomic Energy Research Institute, Jeongeup-si, Republic of Korea; ^2^College of Pharmacy, Chungnam National University, Daejeon, Republic of Korea; ^3^Honam National Institute of Biological Resources, Mokpo-si, Republic of Korea; ^4^College of Pharmacy and Inje Institute of Pharmaceutical Sciences and Research, Inje University, Gimhae-si, Republic of Korea

**Keywords:** *Triticum turgidum* ssp. *turanicum*, benzoxazinone, flavonoid *C*-glycoside, policosanol, UHPLC–QTOF MS, GC–MS, metabolomics, feature-based molecular networking

## Abstract

**Background:**

Oriental wheat (Khorasan wheat; *Triticum turgidum* ssp. *turanicum*; Poaceae) is a tetraploid wheat species that has gained recognition as a superfood due to its high fiber content and nutrient density. Despite its nutritional benefits, limited information is available regarding the metabolite profiles of its sprouts, particularly across different cultivars.

**Methods:**

In this study, metabolite profiles of sprouts from four Oriental wheat cultivars, obtained from the Rural Development Administration’s Genebank, were investigated. An ultra-high performance liquid chromatography–time-of-flight mass spectrometry (UHPLC–QTOF MS) method was employed to analyze and annotate the compounds present. Principal component analysis (PCA) and orthogonal partial least squares discriminant analysis (OPLS-DA) were utilized to explore the metabolite differences among the cultivars. Additionally, feature-based molecular networking analysis was conducted to support metabolite identification and contribute to marker discovery. Gas chromatography–mass spectrometry (GC–MS) was used to quantify policosanols in the samples.

**Results:**

Fourteen compounds were annotated, with eight being detected in T. turgidum ssp. turanicum for the first time. PCA score plots and loading plots revealed distinct metabolite differences among the cultivars based on their country or region of collection. OPLS-DA score plots and *S*-plots indicated the differential expression of five flavonoid *C*-glycosides in samples from Afghanistan and five nitrogen-containing compounds characteristic of samples from Türkiye. The total policosanol content ranged from 356.3 to 400.1 mg/100 g, marking the first quantification of policosanols in Oriental wheat using GC–MS.

**Conclusion:**

These findings provide valuable insights into the phytochemical metabolism of Oriental wheat sprouts and illustrate the influence of geographical conditions on metabolite profiles. The study highlights the potential of Oriental wheat sprouts as a valuable dietary source of policosanols.

## 1 Introduction

Oriental wheat (*Triticum turgidum* ssp. *turanicum*; Poaceae), also known as Khorasan wheat or by its trademarked name Kamut®, is one of the ancient grains mainly cultivated in Asia, capable of growing in diverse climatic conditions (1). These ancient grains are rich in protein and essential minerals such as selenium, zinc, and magnesium, while containing less gluten than conventional wheat varieties (2, 3). They also high in dietary fiber, phenolic acids, tocopherols, and carotenoids (4–7), with a particularly notable antioxidant capacity attributed to their elevated phenolic acid and total polyphenol content (3, 6, 8). Due to these nutritional attributes, the ancient grains have received significant attention for their digestive health and antioxidant benefits. In addition, the medicinal properties of ancient grains have also been highlighted (7, 9–13): Quinoa has shown potent inhibitory effects on α-glucosidase and α-amylase, contributing to control postprandial blood sugar levels and prevent diabetes (7). The consumption of Oriental wheat has been linked to improvements in lipid and glucose profiles, decreased insulin-induced reactive oxygen species (ROS) production, and reduced inflammatory mediators (9). In non-celiac gluten-sensitive patients, Oriental wheat mitigated inflammatory chemokine hyperactivation in peripheral blood mononuclear cells (10). The consumption of Oriental wheat led to reduced serum cholesterol and glucose levels, along with lower ROS and lipid peroxidation, in patient with acute coronary syndrome (11). Oriental wheat improved symptoms in irritable bowel syndrome (IBS) and suppressed associated inflammatory markers (12). It has also been shown to reduce cardiovascular risk by lowering cholesterol and glucose levels, improving redox status, increasing serum potassium and magnesium, and reducing pro-inflammatory cytokine levels (13).

Germination is a common processing technique used to enhance the nutritional profile and bioactivity of grains (14). During germination, wheat undergoes significant compositional changes, including increased levels of carbohydrates, proteins, γ-aminobutyric acid (GABA), and fatty acids, alongside a reduction in anti-nutritional factors such as phytates (14). Moreover, germination increases the concentrations of various antioxidants, including polyphenols, phenolic acids, carotenoids, tocopherols, and vitamin C (15–17). These improvements make sprouts highly nutritious and a promising source of functional food ingredients. Study on Kamut® sprouts and their fermented products have revealed notable β-glucan and total polyphenol contents, along with antioxidant and anti-inflammatory activities (18). Nevertheless, limited research has been conducted the detail phytochemical component and biological properties of Oriental wheat sprouts. Therefore, in this study, the metabolite profiles of sprouts from four Oriental wheat cultivars collected from different regions were analyzed using the ultra-high performance liquid chromatography–quadrupole time-of-flight mass spectrometry (UHPLC–QTOF MS), coupled with multivariate statistical analysis.

Policosanols are a group of long-chain aliphatic alcohols primarily derived from plant sources and beeswax (19–23). Polycosanols have attracted attention for their cholesterol-lowering effects, including inhibition of cholesterol biosynthesis, reduction of low-density lipoprotein (LDL) cholesterol, and elevation of high-density lipoprotein (HDL) cholesterol (24, 25). They also exhibit antioxidant properties, reducing oxidative stress and cellular damage (26), and have been suggested to improve blood circulation and lowers blood pressure (27). While the composition and contents of policosanols have been reported in grain sprouts such wheat (19, 28), barely (20), rice (29), and oat (30), data on Oriental wheat sprouts (*T. turgidum* ssp. *turanicum*) remain lacking. Therefore, in this study, we analyzed the policosanols composition and content in four Oriental wheat sprout samples using gas chromatography–mass spectrometry (GC–MS) to evaluate their potential as an alternative dietary source of policosanols.

## 2 Materials and methods

### 2.1 Plant materials

Four Oriental wheat cultivars (IT308132, IT308447, IT311253, and IT330600) were obtained from the Rural Development Administration’s Genebank. IT308132 and IT311253 were collected from Herat and Chakcharan in Afghanistan, respectively, while IT308447 and IT330600 were collected from Kozluk and Tavas in Türkiye, respectively. 200 seeds of each cultivars were individually sown in 50-cell seedling plug tray filled with soil, watered accordingly, and germinated under controlled conditions. The growth environment was maintained in a growth chamber (DS-50TPLH-3Light, Dasol Science, Republic of Korea) set at 22 °C with 60% relative humidity. White LED light (6,000 K) was used to provide a 16-h light/8-h dark photoperiod. The sprouts were harvested 7 days after sowing, freeze-dried, ground in to powder, and stored in polyethylene plastic bags at −80 °C until further analysis.

### 2.2 Preparation of samples of UHPLC–QTOF MS analysis

For UHPLC−QTOF MS analysis, 1 g of each freeze-dried Oriental wheat sprout sample was extracted with 20 mL of 80% ethanol. The extraction was performed by sonication at room temperature for 1 h, followed by centrifugation at 3,000 × *g* for 5 min (VS-5500N, VISION SCIENTIFIC Co., Daejeon, Republic of Korea). The supernatant was filtrated through 0.45 μm polyvinylidene fluoride (PVDF) filter and concentrated using rotary vacuum evaporator (N-1210BV-W, EYELA, Tokyo, Japan). The resulting dried extracts were dissolved in 1 mL of methanol and further filtered through a 0.20 μm PVDF filter. Each extract was then diluted to a final concentration of 500 ppm in methanol for further UHPLC–QTOF MS analysis. Four replicate samples were prepared from each source using the above preparation method.

### 2.3 UHPLC-QTOF MS analysis

Metabolite analysis was performed using a Waters ACQUITY UPLC system (Waters Corporation, Milford, MA, United States) equipped with a binary solvent delivery system, autosampler, and a UV detector. Separation was achieved on a BEH C18 chromatography column (100 mm × 2.1 mm i.d., 1.7 μm particle size, Waters Corporation). UV–vis absorption spectra were recorded online in the range of 200–500 nm during the UHPLC run. The UHPLC system was coupled to a SYNAPT XS QTOF mass spectrometer (Waters Corporation) for metabolite detection. Each sample (1 μL) was injected at a flow rate of 0.4 mL/min. The column temperature was maintained at 40 °C, and the autosampler was set to 15 °C. The mobile phase consisted of 0.1% formic acid in water (A) and 0.1% formic acid in acetonitrile (B), with the following gradient: 0–20.0 min, 3–15% B; 20.0–21.0 min, 15–100% B; 21.0–26.0 min, 100% B; 26.0–26.1 min, 100–3% B; 26.1–30.0 min, 3% B. The mass spectrometer operated in negative ion mode under the following conditions: source temperature, 120 °C; desolvation temperature, 450 °C; capillary voltage, 2.0 kV; cone voltage, 25 V; cone gas flow, 50 L/h; desolvation gas flow (N_2_), 800 L/h; mass scan range, 100–1,200 Da; scan time, 0.5 s. Leucine-enkephalin was used as the lock mass ([M − H]^−^ *m*/*z* 554.2615). Data were collected in high-definition MS^E^ mode (HDMS^E^), where the instrument alternates between two energy states: a low collision energy of 6 eV and a high energy ranging from 20 to 40 eV. This setup allows for the acquisition of precursor and product ion details across a mass range of *m/z* 100–1,200 Da, captured in distinct spectra within a single acquisition cycle.

### 2.4 Multivariate statistical analysis

Raw data were processed using Progenesis QI software, including steps for normalization, peak alignment, and peak selection. The analysis parameters was set to a retention time range of 0–21.0 min and a mass range of 100–1,200 Da. Multivariate statistical analyses, including principal component analysis (PCA), orthogonal partial least squares discriminant analysis (OPLS-DA), and S-plot, were performed using EZ-Info (Version 14.1, Umetrics, Umeå, Sweden).

### 2.5 Feature-based molecular networking

The UHPLC−QTOF MS raw data files were first converted to ABF format using Reifycs Analysis Base File Converter (version 1.3.8802). MS/MS data deconvolution and peak alignment for feature-based molecular networking were performed using MSDIAL (version 4.9.221218) (31). The resulting alignment outputs were exported as a feature quantification TXT file and an MS/MS spectral summary MGF file. These files were then uploaded to the Global Natural Products Social Molecular Networking (GNPS) platform for feature-based molecular networking (32). A metadata file was also included to assign sample groups based on the country of origin (Afghanistan or Türkiye). The following parameters were used to construct molecular networks: precursor ion mass tolerance of 0.02 Da, fragment ion mass tolerance of 0.02 Da, minimum cosine score of 0.7, and a minimum of 6 matched fragment ions. The molecular networking job can be accessed at: https://gnps.ucsd.edu/ProteoSAFe/status.jsp?task=75dc654d47e54fc8a3d7d0a8c5942b5c. The resulting networks were visualized using Cytoscape software (version 3.10.3) (33).

### 2.6 Preparation of GC–MS analysis samples and policosanol standards

For quantification of policosanols, 1 g of each freeze-dried Oriental wheat sprout samples were extracted into 20 mL of hexane by shaking at 24 °C for 1 h. The extracts were filtrated through 0.45 μm PVDF filter and evaporated under vacuum. For silylation of the policosanols, 0.5 mL of chloroform and 0.25 mL of *N*-methyl-*N*-(trimethylsilyl)trifluoroacetamide (MSTFA; Sigma-Aldrich) were added to each hexane extract. These solutions were reacted in a water bath at 50 °C for 15 min, followed by the addition of 0.5 mL of chloroform. The individual policosanol standards used for peak identification—eicosanol (C20-OH), heneicosanol (C21-OH), docosanol (C22-OH), tricosanol (C23-OH), tetracosanol (C24-OH), hexacosanol (C26-OH), heptacosanol (C27-OH), octacosanol (C28-OH), and triacosanol (C30-OH)—were purchased from Sigma-Aldrich (St. Louis, MO, USA). These standards were also derivatized using MSTFA to produce their trimethylsilane (TMS) derivatives for GC–MS analysis. Calibration curves were constructed using standard solutions at four concentrations (5, 10, 20, and 50 μg/mL). The relationships between the peak areas (𝑦) and concentrations (*x*, μg/ml) were determined using second-order polynomial regression equations, and the correlation coefficients (*R*^2^) were as follows: Eicosanol: *y* = 99,214*x* − 11,360 (*R*^2^ = 0.9963), Heneicosanol: *y* = 85,912*x* + 8119.7 (*R*^2^ = 0.9944), Docosanol: *y* = 40,490*x* − 54,340 (*R*^2^ = 0.9924), Tricosanol: *y* = 66,422*x* − 51,133 (*R*^2^ = 0.9992), Tetracosanol: *y* = 77,881*x* − 87,954 (*R*^2^ = 0.9985), Hexacosanol: *y* = 63,815*x* − 125,820 (*R*^2^ = 0.9914), Heptacosanol: *y* = 66,134*x* − 142,062 (*R*^2^ = 0.9912), Octacosanol: *y* = 59,452*x* − 102,460 (*R*^2^ = 0.994), Triacosanol: *y* = 39,712*x* − 91,914 (*R*^2^ = 0.9925).

### 2.7 GC–MS analysis

The GC–MS analysis was conducted using a Nexis GC-2030 system (Shimadzu, Kyoto, Japan) coupled with a GCMS-QP2020 NX single quadrupole mass spectrometer (Shimadzu). Separation was achieved on an HP-5 MS capillary GC column (30 m × 0.25 mm i.d., 0.25 μm film thickness; Agilent Technologies Co., Santa Clara, CA, USA), with high-purity helium (99.99%) as the carrier gas at a flow rate of 1.2 mL/min. Each sample (1 μL) was injected into the injection port in split mode with 1:5 ratio. The oven temperature was initially set at 230 °C and then ramped to 260 °C at a rate of 25 °C/min, followed by a 10-min hold at 260 °C. The transfer line temperature was maintained at 280 °C. Mass spectrometry data were acquired in electron ionization (EI) mode with an ionization energy of 70 eV, ion source temperature of 230 °C, and scan range of *m/z* 50–500. Collected MS spectra were analyzed using the National Institute of Standards and Technology (NIST) Mass Spectra Library (Gaithersburg, MD, USA). Policosanols were identified by comparing both retention times and the fragmented masses with those of authentic standards.

## 3 Results and discussion

### 3.1 Annotation of metabolites in oriental wheat sprouts

#### 3.1.1 Annotation of metabolites in Oriental wheat sprout samples using UHPLC-QTOF MS

**Figure 1:**
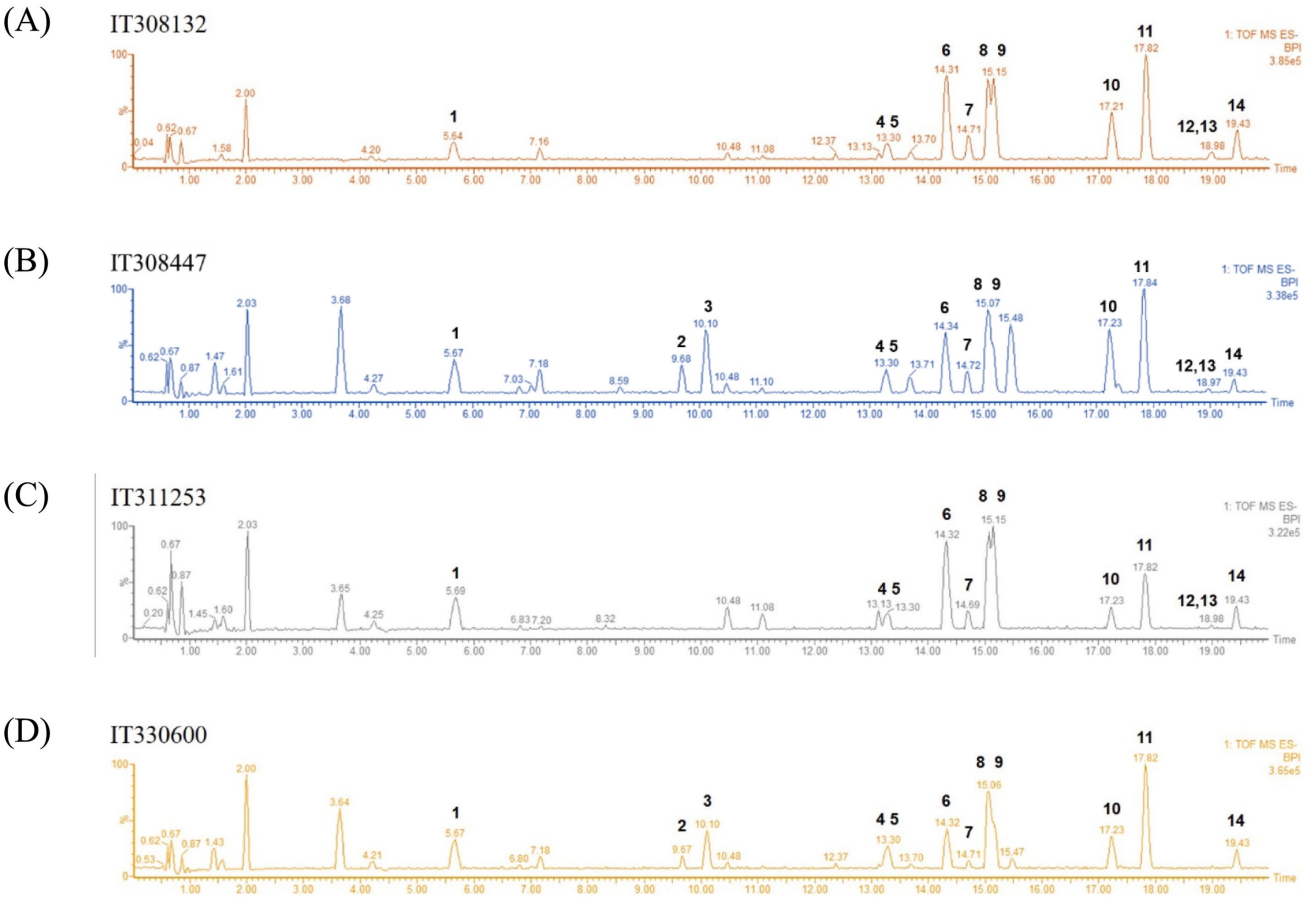
LC–MS base peak ion (BPI) chromatograms of the sprouts of four Oriental wheat cultivars in negative ion mode **(A–D)**. The selected chromatographic peaks are annotated with peak numbers referred to Table 1.

Oriental wheat sprout samples were prepared with four replicates from each source (IT308132, IT308447, IT311253, or IT330600), along with a blank sample. Metabolites in the sprouts of four Oriental wheat cultivars were tentatively characterized using UHPLC−QTOF MS in negative ion mode. The base peak ion (BPI) chromatograms of the 80% ethanol extracts from the Oriental wheat sprout samples are shown in Figure 1. Although the BPI chromatograms displayed a large number of peaks, fourteen metabolites were annotated based on their mass spectra. The annotation was achieved by analyzing retention times and fragmentation patterns of major molecular ions and comparing them with previously reported data. Table 1 presents the retention time (*t*_R_), calculated and observed deprotonated molecular ion *m*/*z* values ([M–H]^−^), calculated mass error (ppm), proposed molecular formulas, and characteristic MS/MS fragment ions for each annotated compound.

**Table 1:** The annotation of metabolites in four Oriental wheat sprouts by UHPLC–QTOF MS.

Peak No.	*t*_R_ (min)	Observed ions [M–H]^−^ (m/z)	Calculated ions [M–H]^−^ (m/z)	Error (ppm)	Molecular formula	Key MS^E^ fragment ions (m/z)	Annotation	Structure	Ref.
1	5.6	371.0974	371.0978	0.6	C_16_H_20_O_10_	193	Dihydroferulic acid-4-*O*-glucuronide	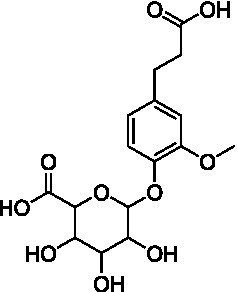	(36)
2	9.6	356.0985	356.0982	0.5	C_15_H_19_NO_9_	194	HMBOA-glucoside	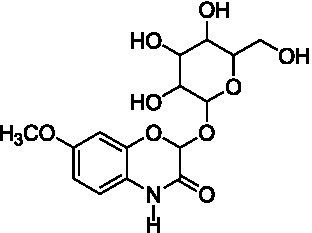	(37)
3	10.1	372.0934	372.0931	0.5	C_15_H_19_NO_10_	210, 164, 149	DIMBOA-glucoside	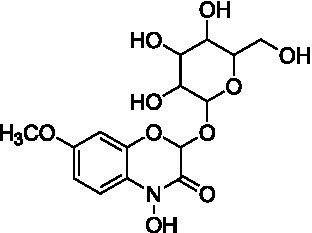	(37)
4	13.1	579.1351	579.1350	0.2	C_26_H_28_O_15_	519, 489, 459, 399	Luteolin-6-*C*-arabinoside-8-*C*-glucoside (isocarlinoside)	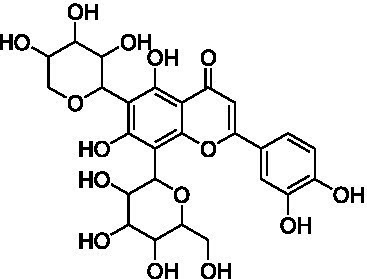	(38)
5	13.3	579.1352	579.1350	0.3	C_26_H_28_O_15_	519, 489, 459, 399	Luteolin-6-*C*-glucoside-8-*C*-arabinoside (carlinoside)	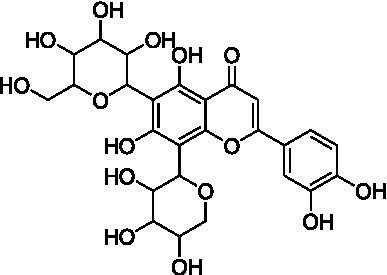	(38)
6	14.3	447.0938	447.0927	2.5	C_21_H_20_O_11_	429, 357, 327, 285	Luteolin-6-*C*-glucoside (isoorientin)	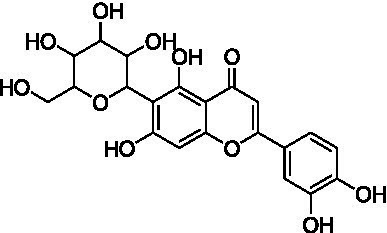	(40)
7	14.7	579.1356	579.1350	1.0	C_26_H_28_O_15_	459, 429, 357	Luteolin-8-*C*-glucoside-2″-*O*-arabinopyranoside (adonivernith)	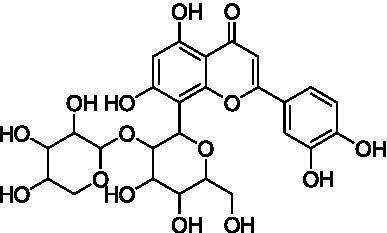	(38)
8	15.0	563.1414	563.1401	2.3	C_26_H_28_O_14_	503, 473, 443, 383, 353	Apigenin-6-*C*-glucoside-8-*C*-arabinoside (schaftoside)	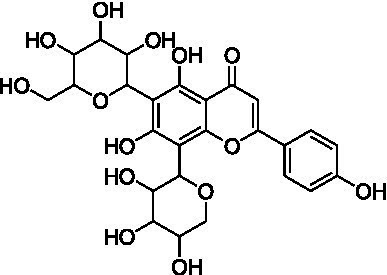	(43)
9	15.2	593.1514	593.1506	1.3	C_27_H_30_O_15_	503, 473, 383, 353	Apigenin 6,8-di-*C*-glucoside (vicenin II)	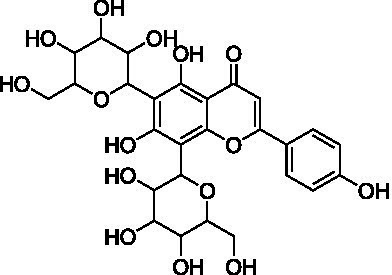	(43)
10	17.2	431.0985	431.0978	1.6	C_21_H_20_O_10_	341, 311	Apigenin-8-*C*-glucoside (vitexin)	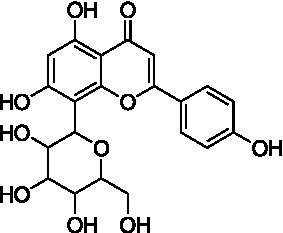	(40)
11	17.8	577.1571	577.1557	2.4	C_27_H_30_O_14_	457, 413, 341	Apigenin 8-*C*-glucoside-2″-*O*-rhamnoside	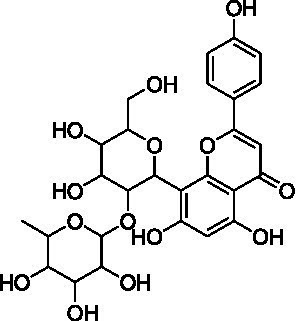	(43)
12	19.0	461.1088	461.1084	0.9	C_22_H_22_O_11_	371, 341	Chrysoeriol-8-*C*-glucoside (scoparin)	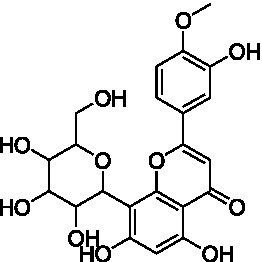	(45)
13	19.0	593.1503	593.1506	−0.5	C_27_H_30_O_15_	443, 371, 341	Chrysoeriol-8-*C*-glucoside-2″-*O*-arabinoside	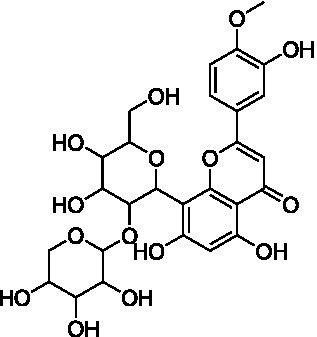	(45)
14	19.4	607.1667	607.1663	0.7	C_28_H_32_O_15_	487, 443, 371	Chrysoeriol-8-*C*-glucoside-2″-*O*-rhamnoside	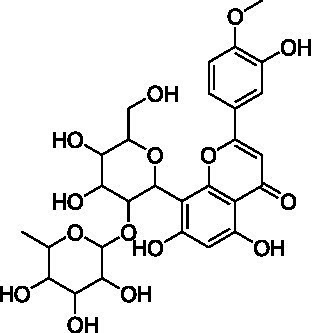	(48)

Among the annotated metabolites, one phenolic acid and two benzoxazinone derivatives were tentatively characterized. These compounds and their glycosides have been previously reported in various cereal crops such as corn, rye, wheat, and adlay (34–36). Notably, DIMBOA [2,4-dihydroxy-7-methoxy-2*H*-1,4-benzoxazin-3(4*H*)-one] and its derivatives are known to contribute allelopathy, insect resistance, and herbicide tolerance (34). Their glycosylated forms are generally considered to be relatively non-toxic constituents in plants (35).

The MS analysis of peak 1 (*t*_R_ = 5.6 min, mass error = 0.6 ppm) revealed a molecular ion [M – H]^−^ at *m*/*z* 371.0974. The MS/MS spectrum displayed a fragment ion at *m*/*z* 193.0504, corresponding to the loss of a glucuronide moiety (−178 Da) ([M – H – 178]^−^) (Supplementary Figure 1). Based on comparison with previously reported MS data, this compound was annotated as dihydroferulic acid-4-*O*-glucuronide (36). This metabolite has been previously reported in wheat (*T. aestivum*) but has not been found in Oriental wheat (*T. turgidum* ssp. *turanicum*).

Peak 2 (*t*_R_ = 9.7 min, mass error = 0.5 ppm) produced a deprotonated molecular ion at *m/z* 356.0985 [M − H]^−^. The MS/MS spectrum showed a fragment ion at *m/z* 194.0461 [M – H – Glu]^−^, consistent with the loss of glucoside moiety (−162 Da) and indicative of HMBOA [2-hydroxy-7-methoxy-2*H*-1,4-benzoxazin-3(4*H*)one] (Supplementary Figure 2). Based on this fragmentation pattern and comparison with reported data, this peak was annotated as 2-(glucopyranosyloxy)-7-methoxy-2*H*-1,4-benzoxazin-3(4*H*)one (HMBOA-glucoside) (37), a compound previously detected in grains of durum wheat (*T. durum*, cv. Kamut).

Peak 3 (*t*_R_ = 10.1 min, mass error = 0.5 ppm) exhibited a major molecular ion at *m/z* 372.0934 [M − H]^−^ (Supplementary Figure 3). The MS/MS fragmentation pattern included: *m/z* 210.0417 [M – H – Glu]^−^, representing the loss of a glucoside moiety (−162 Da), producing DIMBOA [2,4-dihydroxy-7-methoxy-2*H*-1,4-benzoxazin-3(4*H*)-one]; *m/z* 164.0352 [M – H – Glu – OH]^−^, corresponding to an additional loss of a hydroxyl group; and, *m/z* 149.0135 [M – H – Glu – OH – HCOH]^−^, attributed to a further loss of a formyl group (HCOH), producing MBOA (6-methoxy-benzoxazolin-2(3*H*)-one) (34). Based on the these spectral features and comparison with published literature (37), peak 3 was tentatively assigned as 2-(glucopyranosyloxy)-4-hydroxy-7-methoxy-2*H*-1,4-benzoxazin-3(4*H*)-one (DIMBOA-glucoside), a metabolite also reported in grains of durum wheat (*T. durum*, cv. Kamut).

Eleven flavonoids were annotated from Oriental wheat sprout samples. In plants, flavonoids typically accumulate as *O*-glycosylated derivatives; however, in *Triticum* species, the accumulation of flavonoid *C*-glycosides has also been reported (38). In MS/MS fragmentation, *O*-glycosylated flavonoids characteristically lose the sugar moiety via cleavage of the *O*-glycosidic bond, resulting in fragment ions corresponding to aglycones. In contrast, *C*-glycosylated flavonoids exhibit distinct fragmentation patterns due to the breakage of the carbon–carbon (C–C) bond between the aglycon and the sugar moiety (39). Mono-C-glucosyl flavonoids (6-C- or 8-C-isomers) typically show characteristic ions such as [Ag + 41]^−^ and [Ag + 71]^−^; additionally, the [M – H – H₂O]^−^ ion is exclusively detected in 6-*C*-isomers under negative ion mode. Di-*C*-glycosyl flavonoids display the characteristic ions at [Ag+83]^−^ and [Ag+113]^−^. For di-*C*-hexosyl flavonoids, fragment ions at [M – H – 90]^−^ and [M – H – 120]^−^ are commonly observed, whereas 6-*C*-hexosyl-8-*C*-pentosyl-flavonoids typically exhibit fragment ions at [M –H – 60]^−^, [M – H – 90]^−^, and [M–H–120]^−^ (40). It has been suggested that 6-*C*-hexosyl-8-*C*-pentosyl-flavonoids tend to show a strong [M – H – 120]^−^ ion, whereas 6-*C*-pentosyl-8-*C*-hexosyl flavonoids exhibit a higher intensity for the [M – H – 90]^−^ ion (41). For *O*-glycosyl-*C*-glycosyl flavonoids, fragment ions at [M – H − 164]^−^ and [M – H − 150]^−^ indicate the loss of hexose and pentose residues, respectively, characteristic of *O*-glycosylation at phenolic hydroxyl positions or 2″-*O*-glycosylated-*C*-glycosyl derivatives. Moreover, fragment ions at [M–H–hexose–120]^−^ and [M – H – pentose − 120]^−^ are indicated of 6″-*O*-glycosylated-*C*-glycosyl flavonoids (or those glycosylated at the 5″–3″ carbon positions) (42).

The MS analysis of peak 4 (*t*_R_ = 13.1 min, mass error = 0.2 ppm) showed a major molecular ion at *m/z* 579.1351 [M–H]^−^ (Supplementary Figure 4). The MS/MS spectrum exhibited fragment ions at *m/z* 519.1137 [M – H – 60]^−^, 489.1030 [M – H – 90]^−^, 459.0921 [M – H –120]^−^, 399.0715 [aglycone + 113]^−^, and 369.0607 [aglycone + 83]^−^, consistent with a *C*-hexosyl-*C*-pentosyl-luteolin structure containing both hexose and pentose moieties. The relatively higher intensity of the [M – H – 90]^−^ ion compared to [M – H – 120]^−^ suggested a 6-*C*-pentosyl-8-*C*-hexosyl substitution pattern. Based on fragmentation features and comparison with reported literature (38), peak 4 was annotated as luteolin-6-*C*-arabinoside-8-*C*-glucoside (isocarlinoside), a flavonoid previously reported in *T. aestivum* but not in Oriental wheat (*T. turgidum* ssp. *turanicum*).

Peak 5 (*t*_R_ = 13.3 min, mass error = 0.3 ppm) exhibited the same molecular ion at *m/z* 579.1352 [M − H]^−^ and a fragmentation pattern similar to that of peak 4 (Supplementary Figure 5). However, the relative intensity of the [M – H – 120]^−^ ion was greater than that of the [M – H – 90]^−^ ion, indicating a 6-*C*-hexosyl-8-*C*-pentosyl substitution pattern. Based on these observations and comparison with reference data, this peak was tentatively assigned as luteolin-6-*C*-glucoside-8-*C*-arabinoside (carlinoside) (38), which has also been reported in *T. aestivum* but not in Oriental wheat (*T. turgidum* ssp. *turanicum*).

The MS analysis of peak 6 (*t*_R_ = 14.3 min, mass error = 2.5 ppm) showed a major molecular ion at *m/z* 447.0938 [M − H]^−^ (Supplementary Figure 6). The MS/MS spectrum displayed characteristic fragment ions at: *m/z* 429.0830 [M – H – H_2_O]^−^, *m/z* 357.0615 ([M – H – 90]^−^ or [aglycone + 71]^−^), *m/z* 327.0509 ([M – H – 120]^−^ or [aglycone + 41]^−^), and *m/z* 285.0404 [aglycone – H]^−^, indicating a mono-*C*-glycosylated luteolin. The loss of 18 Da corresponding to [M – H – H_2_O]^−^ is typically observed in 6-*C*-hexosyl flavones but is rarely observed in 8-*C*-hexosyl flavones. Therefore, based on these findings and literature comparisons (40), peak 6 was annotated as luteolin-6-*C*-glucoside (isoorientin), a compound reported in the commercial Kamut® (*T. turgidum* ssp. *turanicum*) as well as various cultivars of *T. turgidum ssp. durum* (5).

Peak 7 (*t*_R_ = 14.7 min, mass error = 1.0 ppm) exhibited a deprotonated molecular ion at *m/z* 579.1356 [M − H]^−^ (Supplementary Figure 7). The MS/MS spectrum showed fragment ions at *m/z* 459.0934 [M – H – 120]^−^, 429.0823 [M – H – 150]^−^, and 357.0609 [aglycone + 71]^−^, suggesting *O*-pentosylation at the 2″ position of the *C*-glucosyl-luteolin backbone. The absence of [M – H – H_2_O]^−^ indicated that the *C*-glucoside moiety was located at the 8 position. The fragment ion at [M – H − pentose]^−^ supports the assignment of *O*-glycosylation to the 2″ position (41). Based on features and comparison with published data, this compound was annotated as luteolin-8-*C*-glucoside-2″-*O*-arabinoside (adonivernith) (38), which was has been previously reported in *T. aestivum* leaves, but not in Oriental wheat (*T. turgidum* ssp. *turanicum*).

Peak 8 (*t*_R_ = 15.0 min, mass error = 2.3 ppm) showed a major molecular ion at *m/z* 563.1414 [M − H]^−^ (Supplementary Figure 8). MS/MS fragmentation revealed five major ions: *m/z* 503.1180 [M – H – 60]^−^, *m/z* 473.1084 [M – H – 90]^−^, *m/z* 443.0977 [M – H – 120]^−^, *m/z* 383.0759 [aglycone + 113]^−^, and *m/z* 353.0661 [aglycone + 83]^−^. These fragments are consistent with a *C*-hexosyl-*C*-pentosyl-apigennin structure. The relatively higher intensity of the [M – H – 120]^−^ ion suggested a 6-*C*-hexosyl-8-*C*-pentosyl substitution pattern. Accordingly, the compound was tentatively assigned as apigenin-6-*C*-glucoside-8-*C*-arabinoside (schaftoside) (43), which has been previously detected in Kamut® (*T. turgidum* ssp. *turanicum*) and other *T. turgidum ssp. durum* cultivars (5).

Peak 9 (*t*_R_ = 15.2 min, mass error = 1.3 ppm) had a deprotonated molecular ion at *m/z* 593.1514 [M − H]^−^ (Supplementary Figure 9). Fragment ions observed in the MS/MS spectrum included: *m/z* 503.1178 [M – H – 90]^−^, *m/z* 473.1085 [M – H – 120]^−^, *m/z* 383.0763 [aglycone + 113]^−^, *m/z* 353.0662 [aglycone + 83]^−^. These finding indicate a di-*C*-glucosyl-apigenin, annotated as apigenin 6,8-di-*C*-glucoside (vicenin II) (43). This compound has been reported in commercial Kamut® (*T. turgidum* ssp. *turanicum*) and other cultivars of *T. turgidum ssp. durum* (5).

Peak 10 (*t*_R_ = 17.2 min, mass error = 1.6 ppm) showed a major molecular ion at *m/z* 431.0985 [M − H]^−^ (Supplementary Figure 10). The MS/MS fragmentation pattern included: *m/z* 341.0663 ([M – H – 90]^−^ or [aglycone + 71]^−^), *m/z* 311.0558 ([M – H – 120]^−^ or [aglycone + 41]^−^). These fragments suggest a mono-*C*-hexosyl-apigenin. The absence of [M – H – H_2_O]^−^ supported the assignment of the glucosyl group to the 8-position. Based on comparison with previous literature, this compound was annotated as apigenin-8-*C*-glucoside (vitexin) (43), also detected in Kamut® (*T. turgidum* ssp. *turanicum*) and various cultivars of *T. turgidum ssp. durum* (5).

Peak 11 (*t*_R_ = 17.8 min, mass error = 2.4 ppm) showed a molecular ion at *m/z* 577.1571 [M − H]^−^ (Supplementary Figure 11). The MS/MS fragmentation patterns include *m/z* 457.1138 [M – H – 120]^−^, 413.0876 [M – H – 146]^−^, and 341.0663 [aglycone + 71]^−^, indicating *O*-deoxyhexocylation at the 2″ position of a *C*-glucosyl-apigenin backbone. The absence of the [M – H − H_2_O]^−^ ion supports a *C*-8-glucosyl substitution. Based on these results and literature comparison, the compound was annotated as apigenin 8-*C*-glucoside-2″-*O*-rhamnoside (43), previously reported in wheat (*T. aestivum*) (44), but not in Oriental wheat (*T. turgidum* ssp. *turanicum*).

Peaks 12 and 13 appeared as nearly co-eluting peaks in the BPI chromatogram, with molecular ions at *m/z* 461.1088 [M − H]^−^ and *m/z* 593.1503 [M − H]^−^, respectively (Supplementary Figures 12, 13). In the extracted ion chromatogram for *m/z* 461.1088, fragment ions at *m/z* 371.0768 ([M − H − 90]^−^ or [aglycone + 71]^−^) and *m/z* 341.0665 ([M − H − 120]^−^ or [aglycone + 41]^−^) were detected, indicating a mono-*C*-hexosyl-chrysoeriol structure. The absence of [M – H – H_2_O]^−^ indicated an 8-*C*-glucosyl substitution. Based on spectral data and literature comparison, peak 12 was annotated as chrysoeriol-8-*C*-glucoside (scoparin) (45), previously found in wheat (*T. aestivum*) but not in Oriental wheat (*T. turgidum* ssp. *turanicum*). For peak 13, the extracted ion chromatogram at *m/z* 593.1503 revealed fragment ions at *m/z* 443.0977 [M – H – 150]^−^, 371.0663 [aglycone + 71]^−^, and 341.0665 [aglycone + 41]^−^, indicating *O*-pentosylation at the 2″ position of the *C*-glucosyl-chrysoeriol. The absence of [M – H − H_2_O]^−^ ion supports a *C*-8-glucosyl substitution. This compound was annotated as chrysoeriol-8-*C*-glucoside-2′′-*O*-arabinoside (45), previously isolated from *Setaria italica* (46) and detected in *Dendrobium offificinale* (47), and is here reported in *Triticum* species for the first time.

Peak 14 (*t*_R_ = 19.4 min, mass error = 0.7 ppm) showed a major molecular ion at *m/z* 607.1667 [M − H]^−^ (Supplementary Figure 14). The MS/MS fragmentation include *m/z* 487.1241 [M – H − 120]^−^, 443.0987 [M – H – 146 – 18]^−^, and 371.0767 [aglycone + 71]^−^, consistent with *O*-deoxyhexosylation at C-2″ of a *C*-glucosyl-chrysoeriol. The absence of [M – H − 18]^−^ confirmed the presence of *C*-8-glucosyl substitution. Based on literature comparison, this compound was annotated as chrysoeriol-8-*C*-glucoside-2″-*O*-rhamnoside (48), previously detected in *Cecropia hololeucaa* (48), and is now reported in *Triticum* species for the first time.

#### 3.1.2 Multivariate statistical analysis

**Figure 2:**
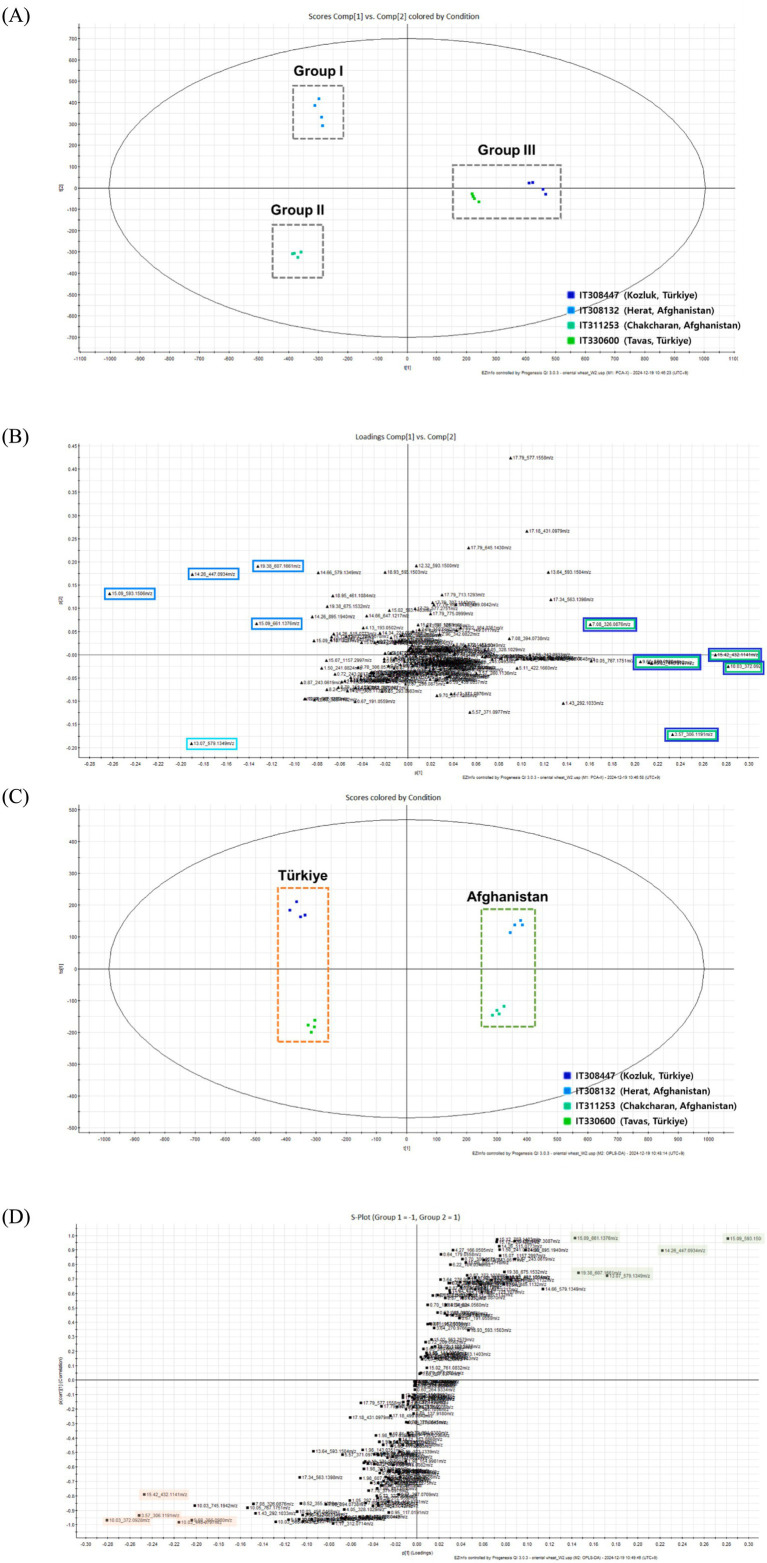
The multivariate analysis of the metabolite data derived from the extract of four Oriental wheat cultivars. **(A)** The score plot and **(B)** loading plot of the PCA model showing PC1-PC2. **(C)** The score plot and **(D)** S-plot of the OPLS-DA model for two plants collected in Türkiye (IT308447 and IT330600) and two plants collected in Afghanistan (IT308132 and IT311253).

To clarify the chemical differences among the sprouts of Oriental wheat cultivars, the metabolite profiles of ethanol extracts from four Oriental wheat sprouts grown under the same environmental conditions were analyzed by UHPLC−QTOF MS. PCA, OPLS-DA, and S-plot, which have been widely used in metabolome analysis of highly complex samples in recent years, are tools to identify significant differences between BPI chromatograms. This serves as an effective approach to compare differences between experimental settings in untargeted and targeted metabolome studies. The four samples were clustered into three groups according to PCA (Figure 2A). Groups I and II were each grouped separately with IT308132 and IT311253 from Afghanistan, while group III consisted of two plants collected in Türkiye, IT308447 and IT330600. The corresponding PCA loading plot showed that ten markers were responsible for group separation (Figure 2B). Four markers (peaks 6, 9, and 14 and an unidentified peak with a molecular ion at *m*/*z* 661.1376, *t*_R_ = 15.09 min) that shifted in the same direction as group I were suggested as distinguishable markers for group I; one marker (peak 4) was distinctive for group II. Five makers aligned with group III, including peaks 2 and 3 and unidentified peaks with molecular ions at *m*/*z* 306.1191 (*t*_R_ = 3.57 min), *m*/*z* 432.1141 (*t*_R_ = 15.42 min), and *m*/*z* 440.0797 (*t*_R_ = 10.03 min). Since the molecular ions of these markers are shown to be even-numbered, they are presumed to contain nitrogen atoms. PCA can identify the overall direction of variation, while OPLS-DA can distinguish variation between specific groups. The OPLS-DA score plot showed that the Oriental wheat sprout samples were clearly separated according to the countries from which they were collected (Figure 2C). The OPLS-DA model produced one predictive and one orthogonal (1 + 3) components, showing a cross-validated predictive ability (*Q*^2^) of 0.988 and a total explained variance (*R*^2^*Y*) of 0.991. In most cases, a *Q*^2^ value greater than 0.5 is considered adequate, and the difference between *R*^2^ and *Q*^2^ should be less than 0.3. The S-plot (point, *t*_R_–*m/z* pair) from the OPLS-DA model, which is a useful tool for comparing the magnitude and reliability of a variables, was also analyzed. The markers, based on the country of collection, were examined according to their distribution in the S-plot (Figure 2D). As with the PCA results, the differential markers of samples collected in Türkiye showed even molecular ions due to the inclusion of nitrogen atoms, as seen in peaks 2 and 3. Conversely, the markers specific to the Afghan samples mainly comprised flavonoid *C*-glycosides, including peaks 4, 6, 9, and 14. Therefore, these results could serve as important references for identifying quality markers in Oriental wheat breeding programs based on country of cultivation.

#### 3.1.3 Feature-based molecular networking analysis

**Figure 3:**
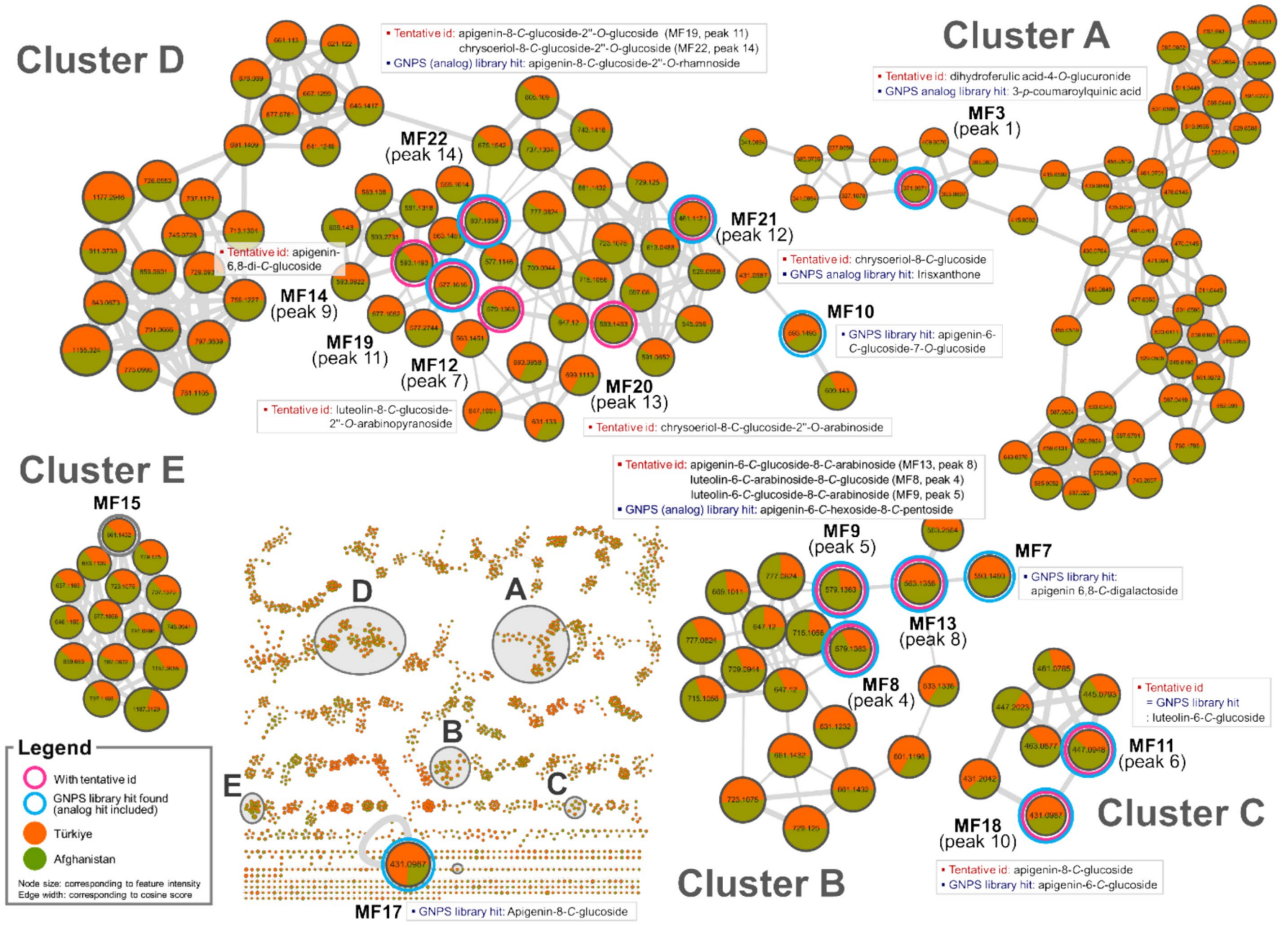
Molecular network clusters A–E of Oriental wheat sprout samples.

**Figure 4:**
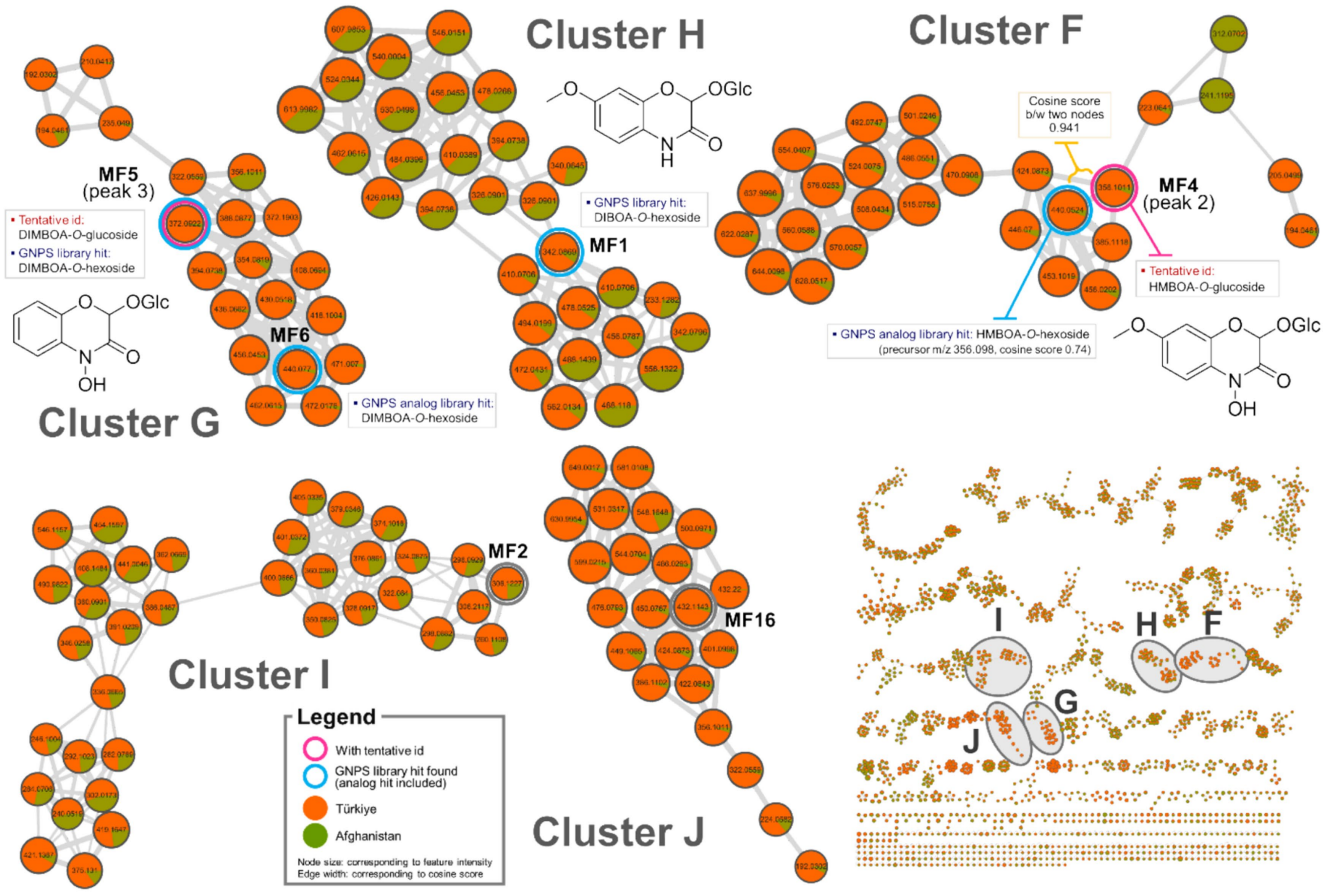
Molecular network clusters F–J of Oriental wheat sprout samples.

Feature-based molecular networking has become a widely used method for analyzing MS/MS data due to its capacity to visualize large datasets based on fragmentation ma similarity and to suggest possible structures using extensive cloud-based libraries. Additionally, it facilitates semi-quantitative comparisons between groups by evaluating differences in MS1 feature intensities (32). In the present study, feature-based molecular networking analysis was employed to reinforce the annotation of chemical markers that distinguish Oriental wheat samples based on their countries of origin, as suggested by multivariate analysis. This method also aimed to uncover additional candidate markers. Feature-based molecular networking of the Oriental wheat sprout samples was conducted with grouping based on collection origin: Türkiye (orange) and Afghanistan (green), as indicated in the pie charts. The results closely aligned with those from the multivariate analysis (Figures 3, 4). Analog-based library searches further enhanced the reliability of metabolite annotation, providing plausible matches for many of the features (Table 2).

**Table 2:** Selected MS^1^ features (MF) of oriental wheat sprouts in GNPS feature-based molecular networking analysis.

MF No.	*t*_R_ (min)	Parent mass (m/z)	GNPS cluster index	GNPS (analog) library hit (precursor m/z, cosine score)	Corresponding peak (tentative id)
1	2.58	342.0869	897	DIBOA-*O*-hexoside (342.082, 0.87)	NA^a^
2	3.62	306.1227	711	NA	NA
3	5.62	371.0971	1,023	Analog: 3-*p*-coumaroylquinic acid (367.103, 0.91)	Peak 1 (dihydroferulic acid-4-*O*-glucuronide)
4	9.64	356.1011	968	NA	Peak 2 (HMBOA-*O*-glucoside)
5	10.07	372.0922	1,033	DIMBOA-*O*-hexoside (372.093, 0.93)	Peak 3 (DIMBOA-*O*-glucoside)
6	10.07	440.0770	1,317	DIMBOA-*O*-hexoside (372.093, 0.89)	NA
7	12.33	593.1493	1774	Apigenin 6,8-*C*-digalactoside (593.151, 0.81)	NA
8	13.12	579.1363	1734	Analog: apigenin-6-*C*-hexoside-8-*C*-pentoside (563.141, 0.82)	Peak 4 (luteolin-6-*C*-arabinoside-8-*C*-glucoside)
9	13.29	579.1363	1735	Analog: apigenin-6-*C*-hexoside-8-*C*-pentoside (563.141, 0.84)	Peak 5 (luteolin-6-*C*-glucoside-8-*C*-arabinoside)
10	13.65	593.1493	1772	Apigenin-6-*C*-glucoside-7-*O*-glucoside (593.149, 0.84)	NA
11	14.28	447.0948	1,337	Luteolin-6-*C*-glucoside (447.093, 0.93)	Peak 6 (luteolin-6-*C*-glucoside)
12	14.67	579.1363	1733	NA	Peak 7 (luteolin-8-*C*-glucoside-2″-*O*-arabinopyranoside)
13	15.03	563.1358	1,680	Apigenin 6,8-*C*-digalactoside (593.151, 0.84)	Peak 8 (apigenin-6-*C*-glucoside-8-*C*-arabinoside)
14	15.11	593.1493	1771	NA	Peak 9 (apigenin-6,8-di-*C*-glucoside)
15	15.12	661.1432	1912	NA	NA
16	15.44	432.1143	1,283	NA	NA
17	16.59	431.0987	1,278	Apigenin-8-*C*-glucoside (431.098, 0.91)	NA
18	17.19	431.0987	1,277	Apigenin-6-*C*-glucoside (431.098, 0.92)	Peak 10 (apigenin-8-*C*-glucoside)
19	17.79	577.1616	1727	Apigenin-8-*C*-glucoside-2″-*O*-rhamnoside (577.156, 0.87)	11 (apigenin 8-*C*-Peak glucoside-2″-*O*-rhamnoside)
20	18.93	593.1493	1773	NA	Peak 13 (chrysoeriol-8-*C*-glucoside-2″-*O*-arabinoside)
21	18.95	461.1121	1,396	Analog: irisxanthone (435.093, 0.78)	Peak 12 (chrysoeriol-8-*C*-glucoside)
22	19.39	607.1659	1815	Analog: apigenin-8-*C*-glucoside-2″-*O*-rhamnoside (577.156, 0.85)	Peak 14 (chrysoeriol-8-*C*-glucoside-2″-*O*-rhamnoside)

a NA: not available for the GNPS (analog) library hit, not applicable for the corresponding peak.

Among the fourteen key peaks annotated from the BPI chromatograms, flavonoid *C*-glycosides constituted the majority of metabolites in the Oriental wheat sprouts. The multivariate analysis identified peaks 4, 6, 9, and 14 as differential markers for Afghanistan samples. In the molecular network, clusters B − D were specifically enriched with flavonoid *C*-glycosides, and the aforementioned peaks corresponded to the following features: MF8 in cluster B, MF11 in cluster C, and MFs 14 and 22 in cluster D (Figure 3). The pie charts visualizations clearly indicated the predominance of these components in the Afghanistan samples, as the green segments were significantly larger than the orange segments. Notably, MF11 (peak 6, cluster C) had a high cosine score (0.93) in the GNPS library, consistent with its annotation as luteolin-6-C-glucoside. Other plausible analogs supported additional annotations: MF8 (peak 4, cluster B) matched apigenin-6-*C*-hexoside-8-*C*-pentoside, which is consistent with the annotation of luteolin-6-*C*-arabinoside-8-*C*-glucoside; MFs 19 and 22 (peaks 11 and 14, respectively, in cluster D) matched apigenin-8-*C*-glucoside-2″-*O*-rhamnoside and chrysoeriol-8-*C*-glucoside-2″-*O*-rhamnoside. Furthermore, three previously unidentified features were assigned as flavonoid *C*-glycosides based on spectral library searches with cosine scores greater than 0.8: MF7 (apigenin 6,8-*C*-digalactoside), MF10 (apigenin-6-*C*-glucoside-7-*O*-glucoside), and MF17 (apigenin-8-*C*-glucoside). However, MF15, despite being a distinguishing feature of the Afghan samples, could not be structurally annotated using the current methods.

The molecular networks also included clusters of feature nodes that were quantitatively more abundant in the Türkiye samples. In the pie charts within these clusters, the orange segments—representing Türkiye—were larger than the green segments—representing Afghanistan. Clusters F, G, and H were interpreted as potentially associated with nitrogen-containing compounds, which were previously suggested as distinguishing markers for the Türkiye samples in the multivariate analysis (Figure 4). The GNPS library search proposed HMBOA-*O*-hexoside and DIMBOA-*O*-hexoside as likely identities for the features in clusters F and G, respectively. MF6, a marker that remained unidentified the multivariate analysis, was found to be clustered with MF5 (corresponding to peak 2 in cluster F, annotated as HMBOA-*O*-glucoside) and also yielded an analog library hit as DIMBOA-*O*-hexoside. Furthermore, MF1, a feature in cluster H, had a library match with another nitrogen-containing compound, DIBOA-O-hexoside, with a cosine score of 0.87. However, for MFs 2 and 16, located in clusters I and J, respectively, no plausible structural annotations were retrieved from the GNPS library search.

### 3.2 The contents of policosanol in the oriental wheat sprout samples

Policosanol compounds in the hexane extracts of the four Oriental wheat sprout samples were analyzed by GC–MS, using the TMS derivatives of policosanol standards. The mass spectra of the respective TMS-derivatized policosanols displayed major ion peaks corresponding to [M − 15]^+^, indicative of the loss of a methyl group (−CH_3_). Based on the GC–MS analysis of the samples, six policosanols—tricosanol (C23-OH), tetracosanol (C24-OH), hexacosanol (C26-OH), heptacosanol (C27-OH), octacosanol (C28-OH), and triacontanol (C30-OH)—were identified, whereas eicosanol (C20-OH), heneicosanol (C21-OH), and docosanol (C22-OH) were not detected (Supplementary Figures 15, 16). Quantitative analysis of the six identified policosanols in each Oriental wheat sprout sample revealed that octacosanol (C28-OH) was the most abundant component across all samples (Table 3). The highest total policosanol content was observed in IT308447 (400.1 mg/100 g), followed by IT311253 (377.4 mg/100 g). However, these two samples showed differing patterns in individual policosanol distribution: IT308447 exhibited higher levels of C24-OH, C26-OH, C27-OH, and C28-OH compared to IT311253, while IT311253 had higher levels of C23-OH and C30-OH. The policosanol profile of IT330600 was similar to that of IT308447. Among the samples, IT308132 had the highest concentration of C30-OH but the lowest level of C28-OH. These findings suggest that policosanol content and composition in Oriental wheat sprouts are not strictly dependent on the country of origin. Octacosanol (C28-OH) found to be the predominant policosanol, consistent with previous findings in common wheat (*T. aestivum* L.) sprouts, including Korean cultivars Geumkang and Cheongwoo (49). In contrast, hexacosanol (C26-OH) has been reported as the major policosanol in other cereals such as barley and oats (20, 30). The average total policosanol content of the four Oriental wheat sprout samples was 376.1 mg/100 g, higher than the average value (345.0 mg/100 g) previously reported for two common wheat cultivars (49). This value also exceeds the policosanol contents reported for other natural sources, including sugarcane (up to 270 mg/100 g), beeswax (up to 12 mg/100 g), adlay (up to 246 mg/100 g), corn kernel (up to 20 mg/100 g), peanut (up to 54 mg/100 g), and winter spinach (up to 59 mg/100 g) (22, 50–53). This study is the first to evaluate both the individual and total policosanol contents of Oriental wheat sprout samples. Given their high policosanol levels, Oriental wheat sprouts may serve as a promising dietary source of policosanols with potential functional benefits.

**Table 3:** Contents of policosanols of the hexane extracts of oriental wheat sprout samples.

Policosanols^a^	Policosanol content (mg/100 g)^b^			
	IT308132	IT308447	IT311253	IT330600
C20–OH	ND^c^	ND	ND	ND
C21–OH	ND	ND	ND	ND
C22–OH	ND	ND	ND	ND
C23–OH	15.5 ± 6.1	10.5 ± 4.3	21.9 ± 8.5	23.6 ± 9.7
C24–OH	4.8 ± 1.9	4.4 ± 2.0	3.7 ± 2.3	2.2 ± 0.7
C26–OH	23.7 ± 9.6	28.7 ± 11.3	23.3 ± 9.6	22.0 ± 9.5
C27–OH	4.3 ± 1.8	5.4 ± 2.3	4.3 ± 1.9	4.0 ± 1.8
C28–OH	264.0 ± 7.9	321.4 ± 8.3	288.2 ± 10.3	287.3 ± 9.4
C30–OH	44.0 ± 20.6	29.7 ± 13.9	35.8 ± 19.4	31.9 ± 18.1
Total	356.3 ± 48.0	400.1 ± 42.1	377.2 ± 51.9	370.9 ± 49.2

a C20, eicosanol; C21, heneicosanol; C22, docosanol; C23, tricosanol; C24, tetracosanol; C26, hexacosanol; C27, heptacosanol; C28, octacosanol; C30, triacontanol; and total PC content.

b All values are the mean ± standard deviation of three independent experiments.

c Not detected.

## 4 Conclusion

The metabolite profiles of Oriental wheat sprout samples collected from Türkiye and Afghanistan were analyzed using UHPLC–QTOF MS, leading to the annotation of 14 compounds, including one phenolic acid, two benzoxazinones, and eleven flavonoid *C*-glycosides. Among them, six compounds were reported for the first time in Oriental wheat (*T. turgidum* ssp. *turanicum*), and two compounds—chrysoeriol-8-*C*-glucoside-2″-*O*-arabinoside and chrysoeriol-8-*C*-glucoside-2″-*O*-rhamnoside—were reported for the first time in the *Triticum* genus. Multivariate statistical analysis revealed clear metabolite distinctions based on the country of origin. Specifically, samples from Türkiye were characterized by nitrogen-containing compounds such as benzoxazinones, while those from Afghanistan were enriched in flavonoid *C*-glycosides. These compositional patterns were corroborated by feature-based molecular networking analysis using MS/MS data, which further supported the differentiation by geographical origin. Additionally, policosanol composition and content in the sprouts of the four Oriental wheat cultivars were assessed using GC–MS. The total policosanol content ranged from 356.3 to 400.1 mg/100 g, which is exceptionally high compared to other known sources. Octacosanol (C28-OH) was identified as the dominant policosanol, accounting for more than 74% of the total content. Overall, this study provides valuable insights into the phytochemical diversity and nutritional potential of Oriental wheat sprouts, supporting their use as a promising source of bioactive compounds with functional food applications.

## Data Availability

The original contributions presented in the study are included in the article/Supplementary material, further inquiries can be directed to the corresponding authors.
