# Supplementary material for: Comparison of metabolite profiles and policosanol contents in the sprout of Oriental wheat cultivars (Triticum turgidum ssp. turanicum)
Source: Front Nutr. 2025 Sep 10;12:1649097. doi: 10.3389/fnut.2025.1649097 (PMC12459278; doi:10.3389/fnut.2025.1649097)
Supplement: Supplementary file 1 [file Supplementary_file_1.DOCX]

Supplementary Material

# Supplementary Figures


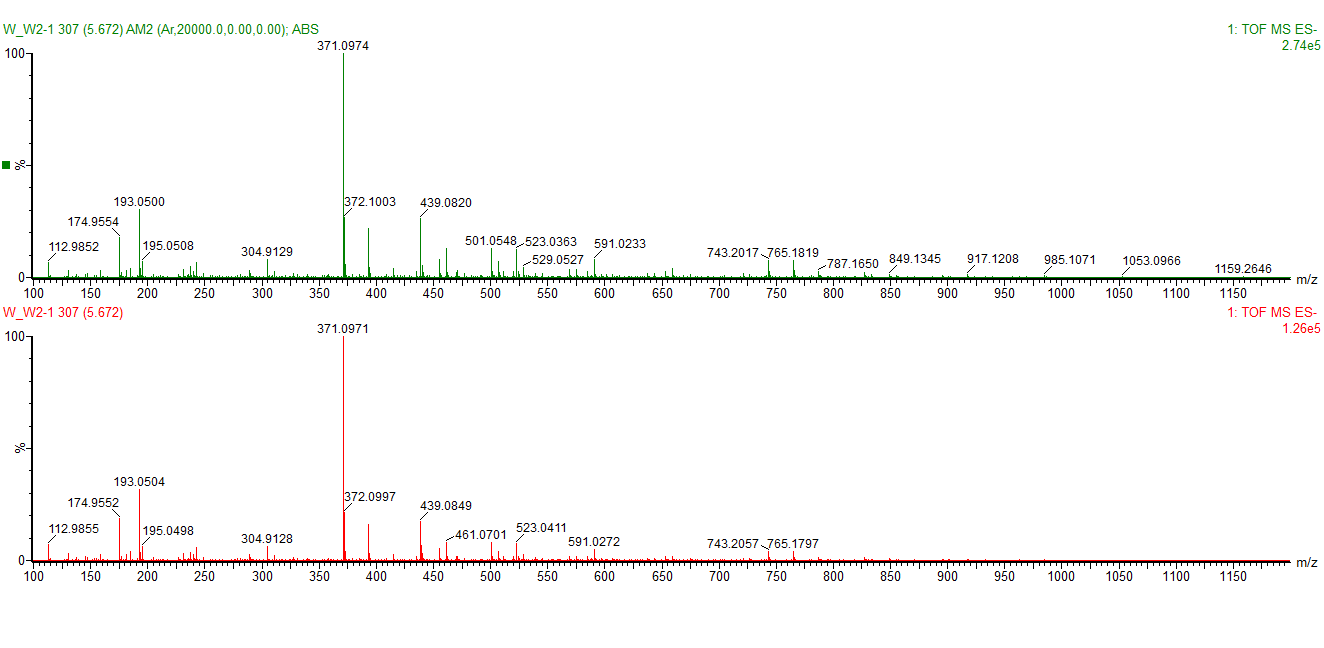


**Supplementary Figure 1.** ESI-QTof-MS spectrum of dihydroferulic acid-4-*O*-glucuronide (peak 1).


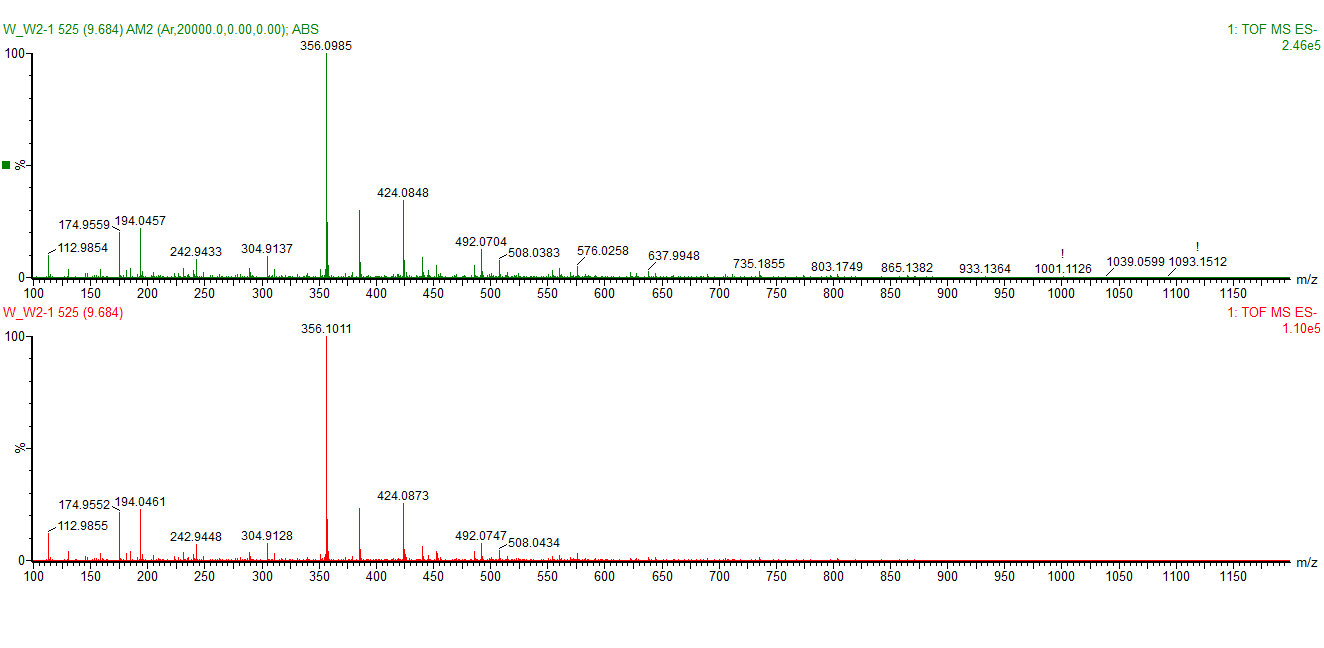


**Supplementary Figure 2.** ESI-QTof-MS spectrum of 2-(glucopyranosyloxy)-7-methoxy-2*H*-1,4-benzoxazin-3(4*H*)-one (peak 2).

**
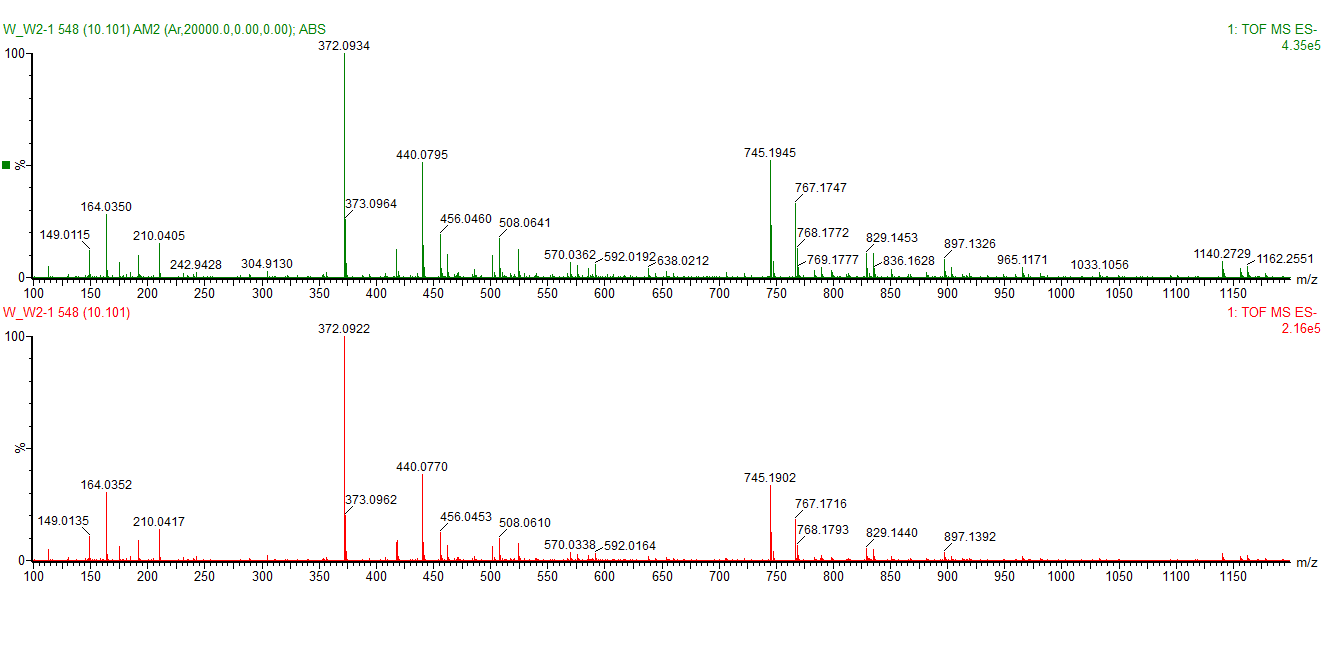
**

**Supplementary Figure 3.** ESI-QTof-MS spectrum of 2-*O*-(glucopyranosyloxy)-4-hydroxy-7-methoxy-2*H*-1,4-benzoxazin-3(4*H*)-one (peak 3).


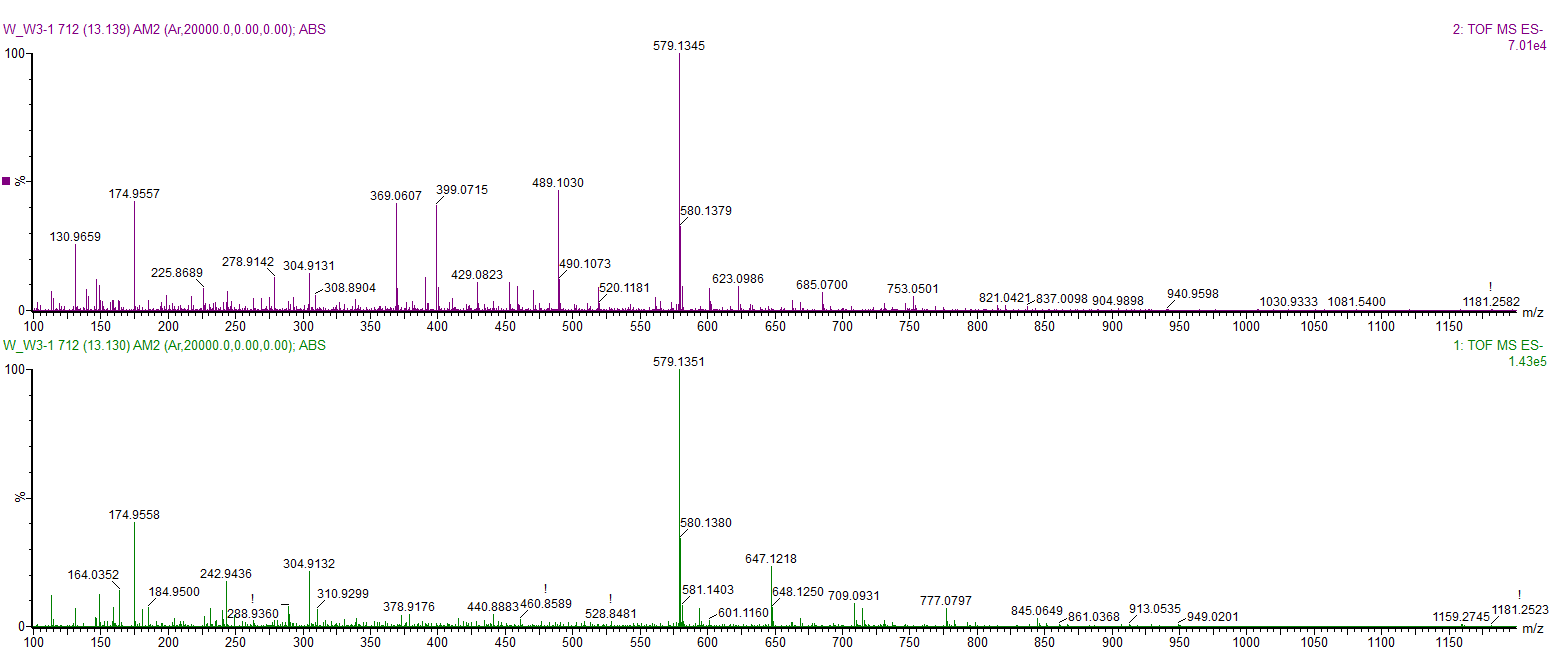


**Supplementary Figure 4.** ESI-QTof-MS spectrum of luteolin-6-*C*-arabinoside-8-*C*-glucoside (peak 4).

**
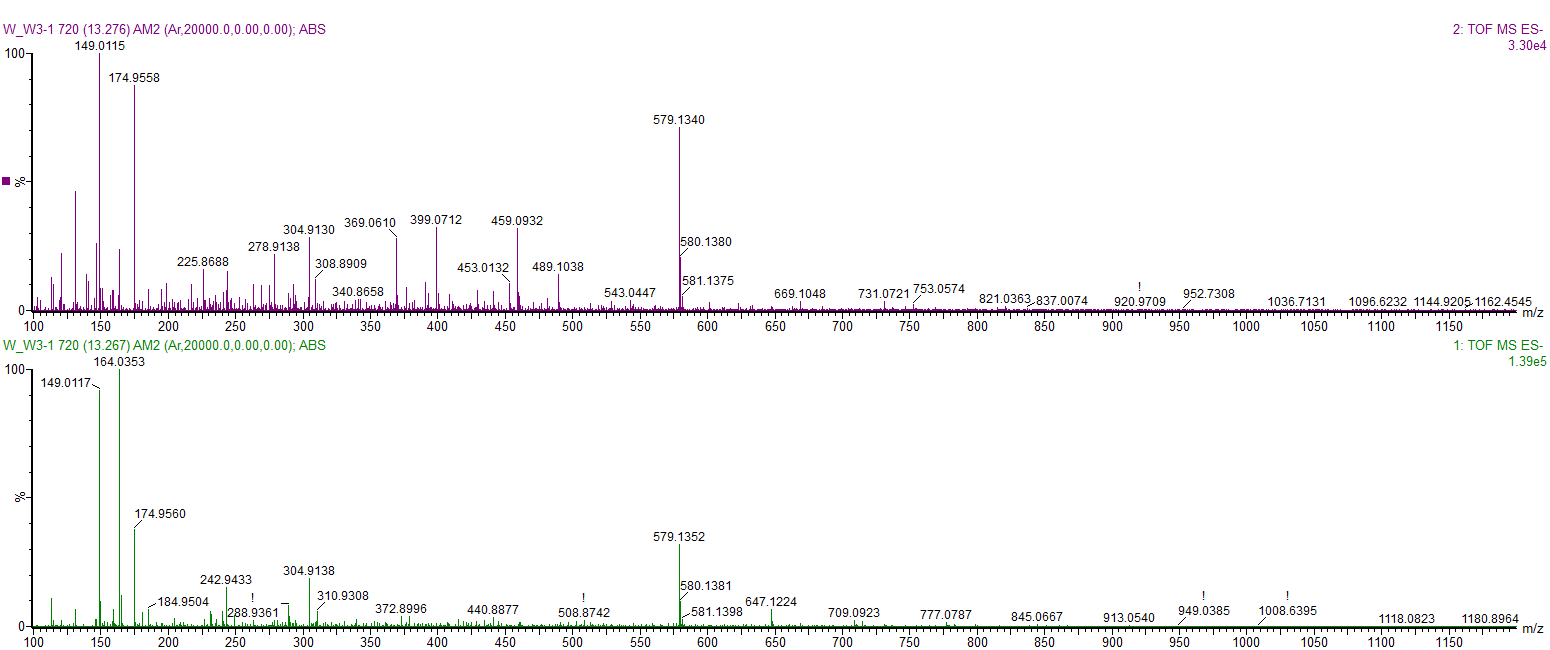
**

**Supplementary Figure 5.** ESI-QTof-MS spectrum of luteolin-6-*C*-glucoside-8-*C*-arabinoside (peak 5).

**
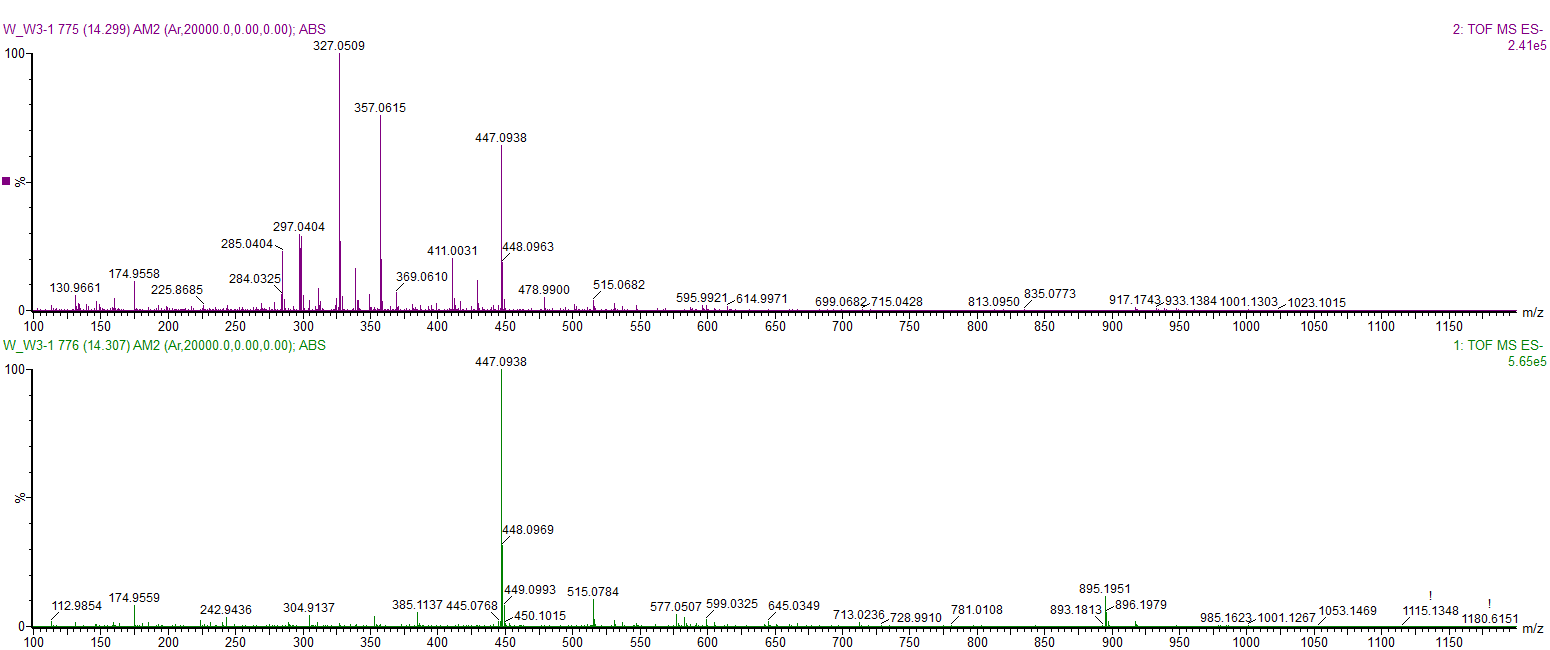
**

**Supplementary Figure 6.** ESI-QTof-MS spectrum of luteolin-6-*C*-glucoside (peak 6).

**
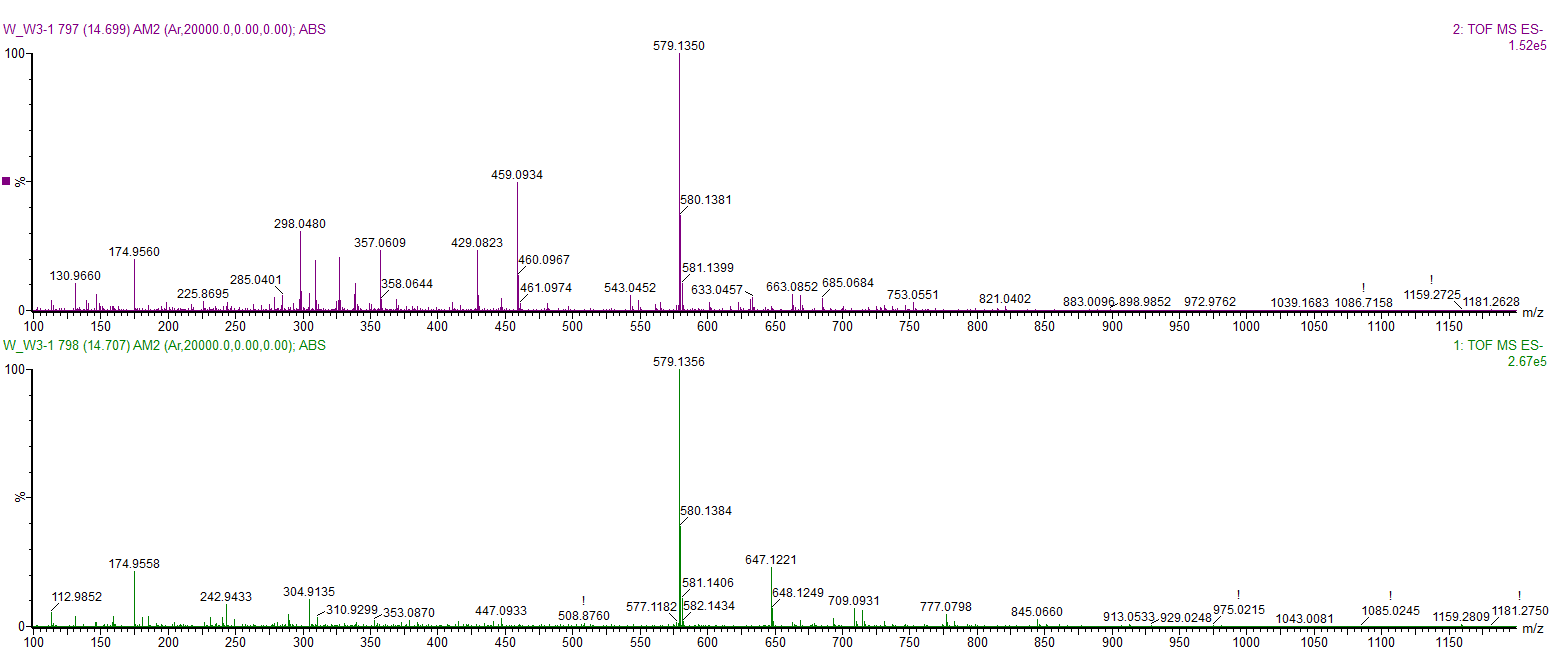
**

**Supplementary Figure 7.** ESI-QTof-MS spectrum of luteolin-8-*C*-glucoside-2′′-*O*-arabinopyranoside (peak 7).

**
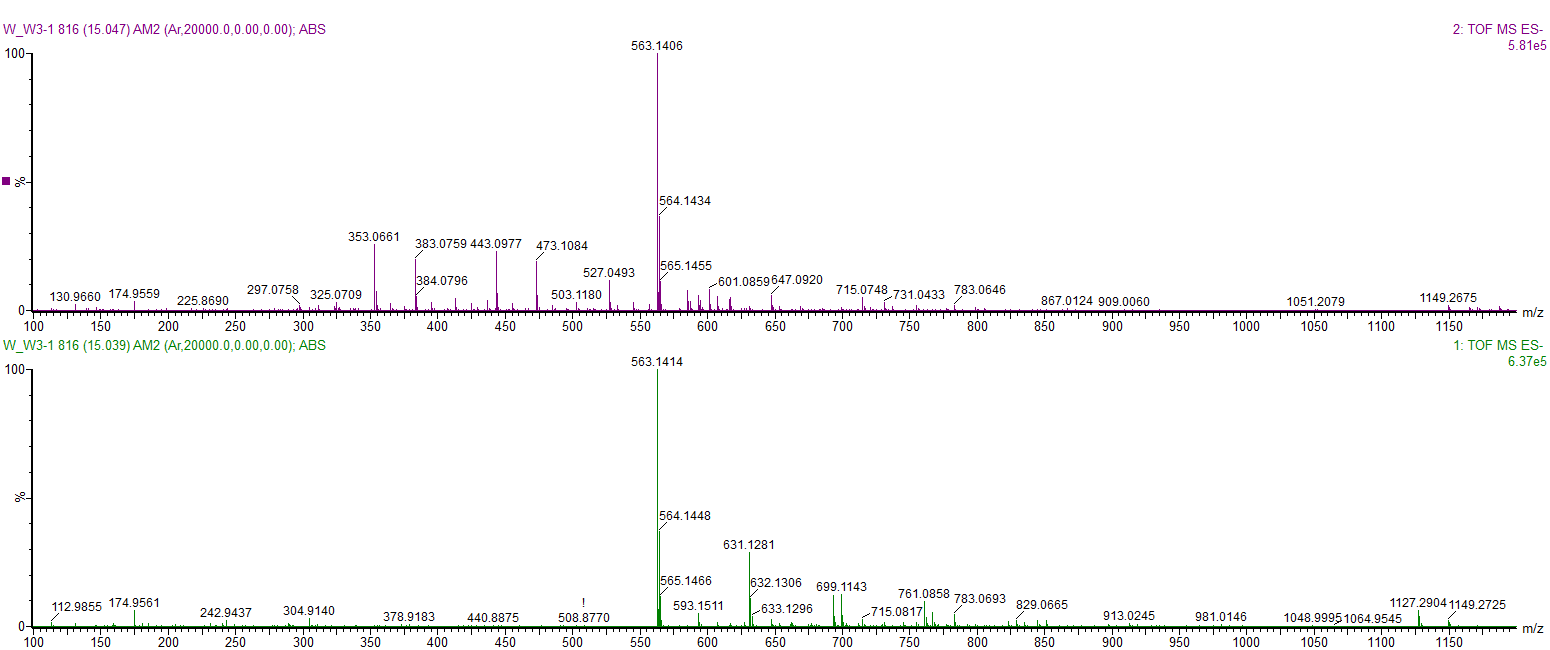
**

**Supplementary Figure 8.** ESI-QTof-MS spectrum of apigenin-6-*C*-glucoside-8-*C*-arabinoside (peak 8).

**
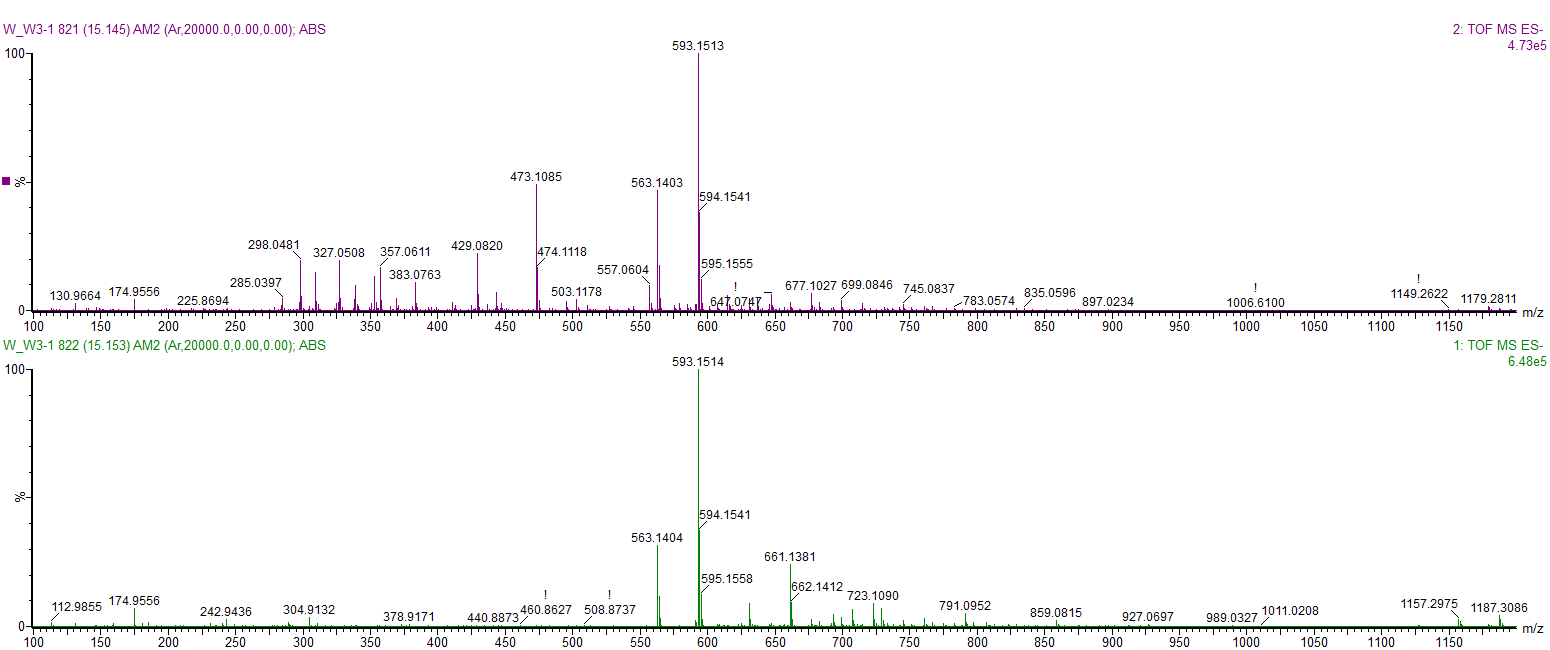
**

**Supplementary Figure 9.** ESI-QTof-MS spectrum of apigenin 6-C-glucoside-8-C-glucoside (peak 9).

**
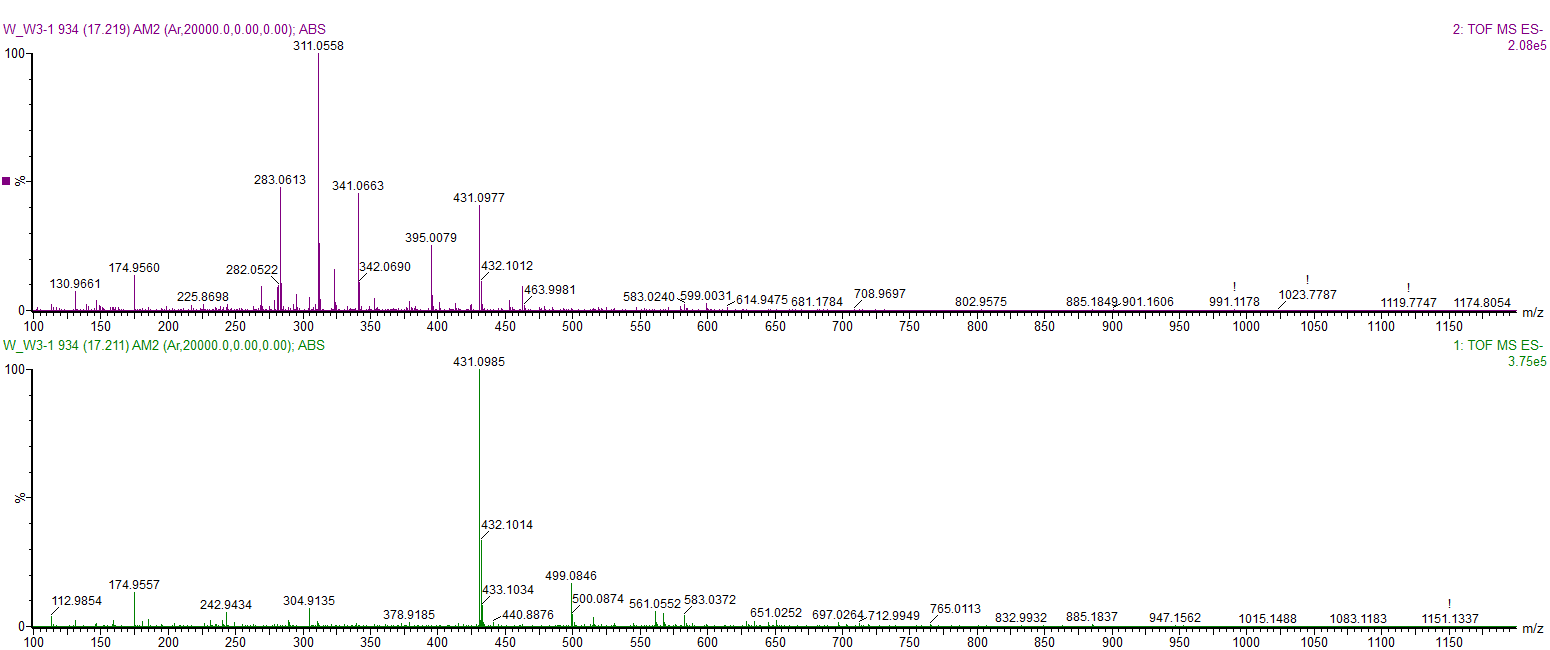
**

**Supplementary Figure 10.** ESI-QTof-MS spectrum of apigenin-8-*C*-glucoside (peak 10).

**
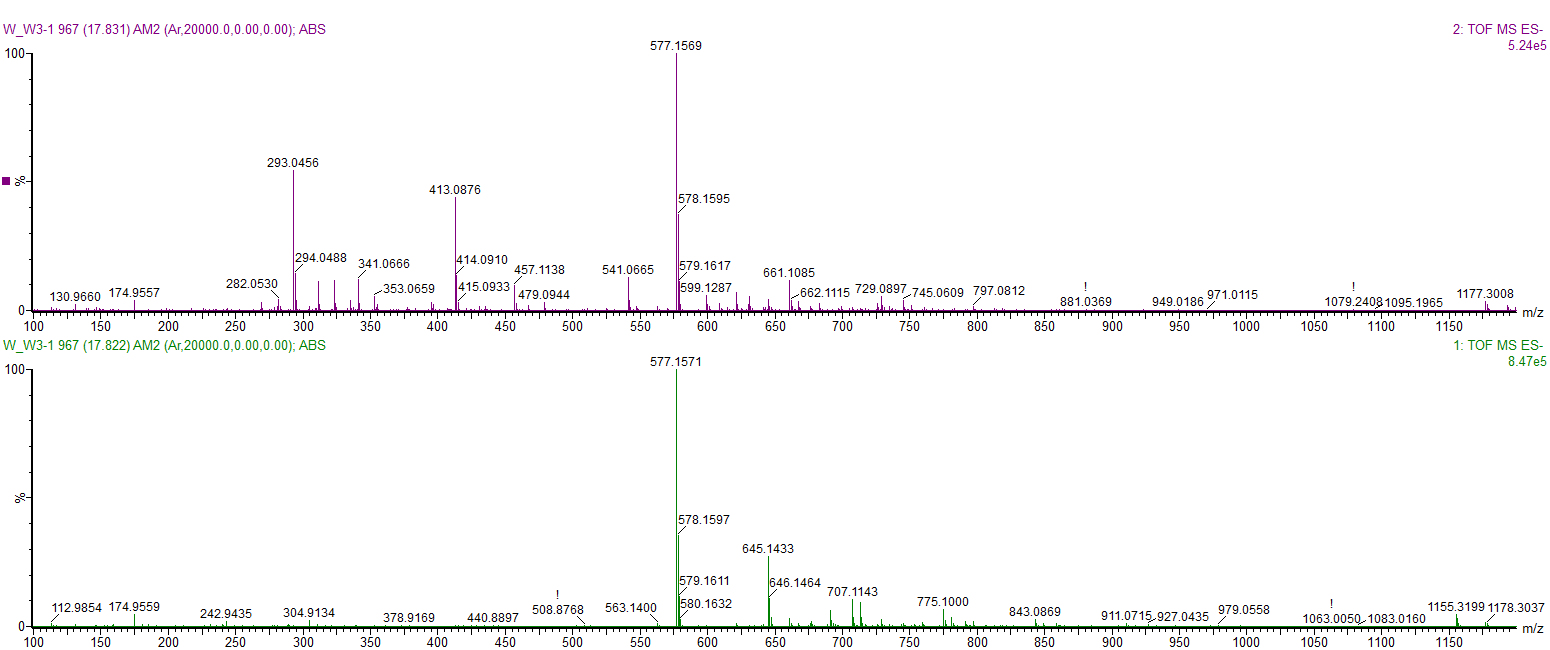
**

**Supplementary Figure 11.** ESI-QTof-MS spectrum of apigenin 8-*C*-glucoside-2''-*O*-glucoside (peak 11).

**
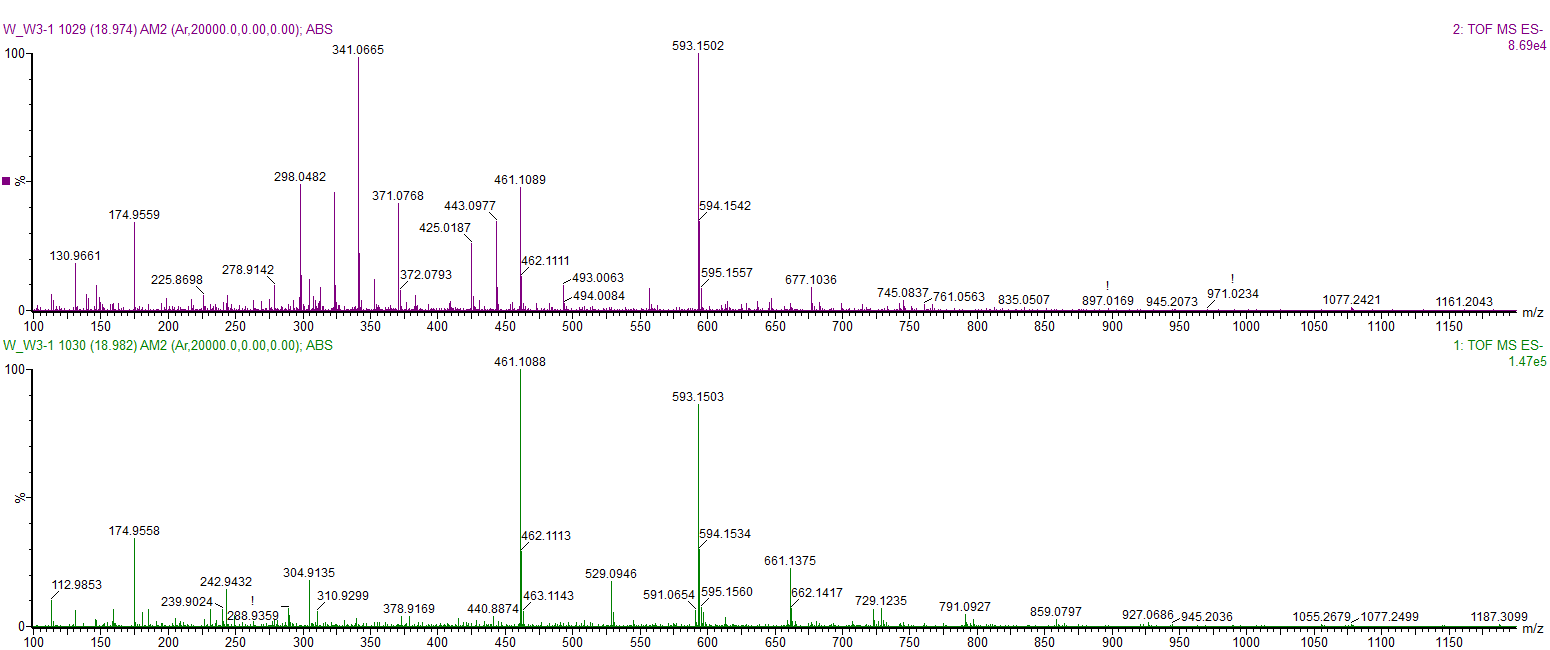
**

**Supplementary Figure 12.** ESI-QTof-MS spectrum of chrysoeriol-8-C-glucoside (peak 12).

**
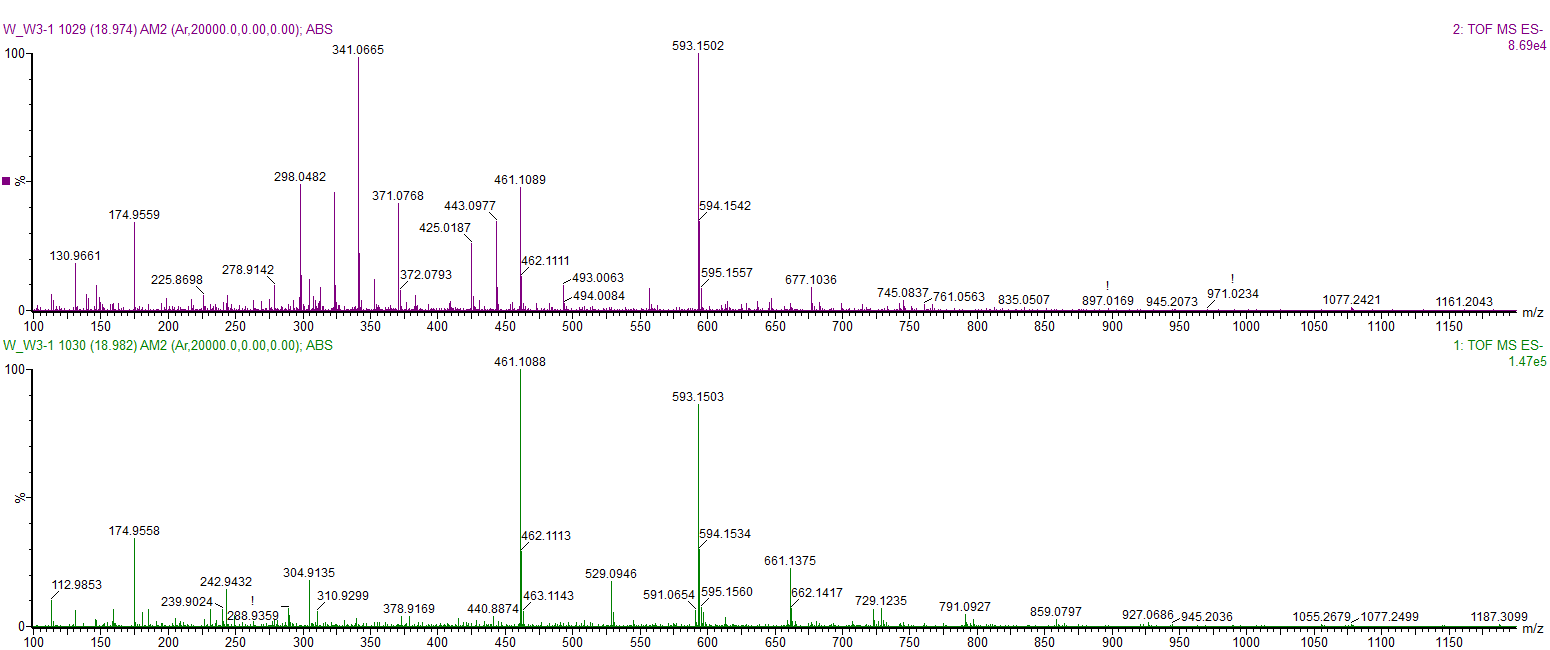
**

**Supplementary Figure 13.** ESI-QTof-MS spectrum of chrysoeriol-8-*C*-glucoside-2′′-*O*-arabinoside (peak 13).

**
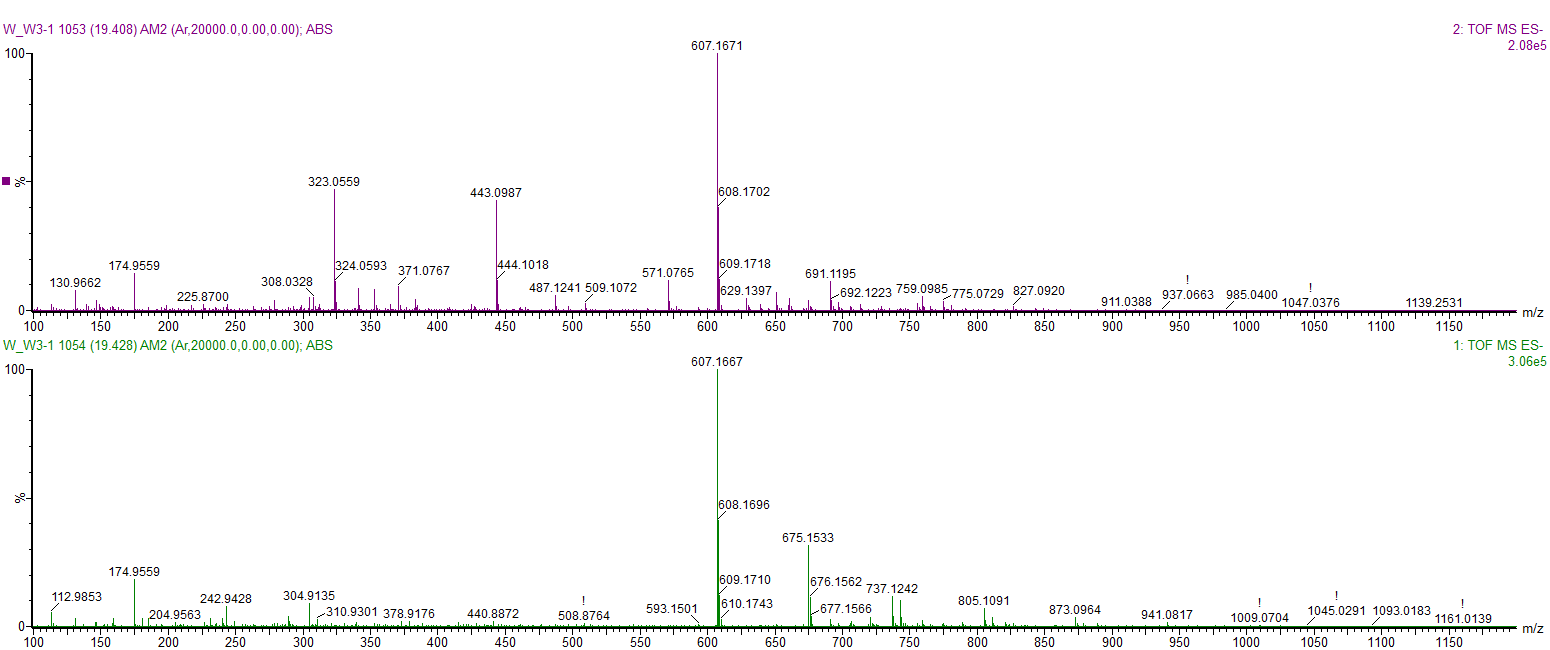
**

**Supplementary Figure 14.** ESI-QTof-MS spectrum of chrysoeriol-8-*C*-glucoside-2′′-*O*-glucoside (peak 14).

**
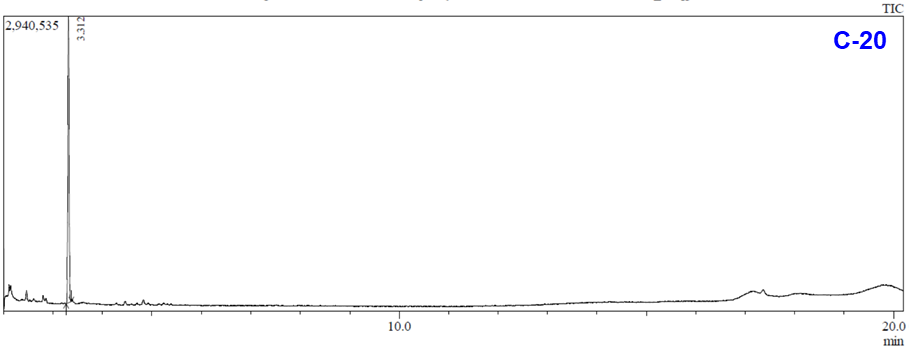
**

**
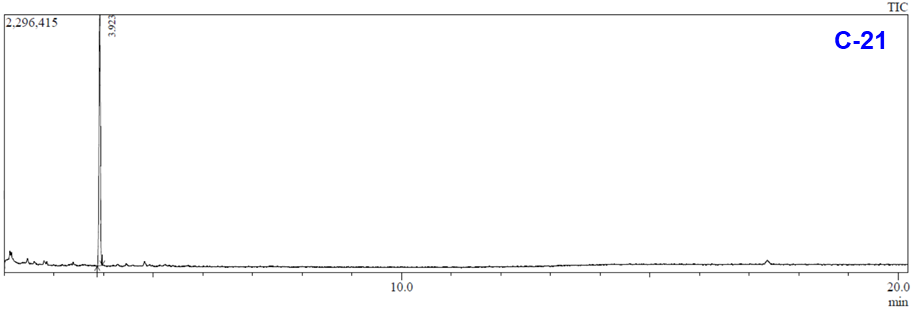
**

**
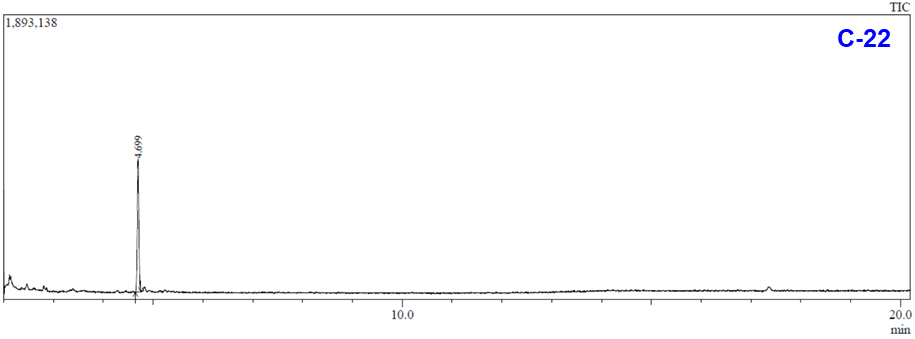
**

**
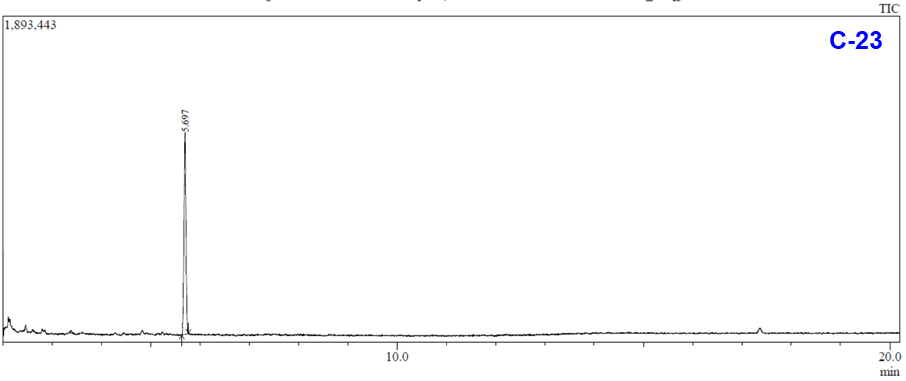
**

**
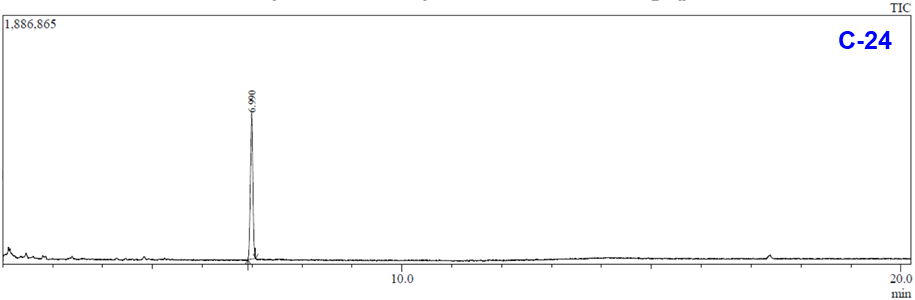
**

**
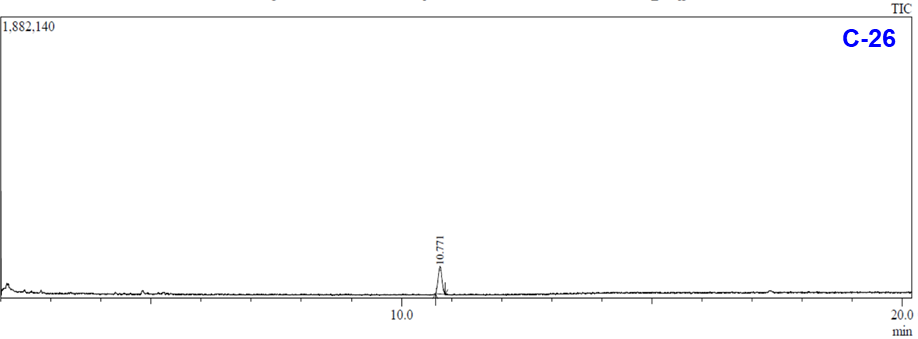
**

**
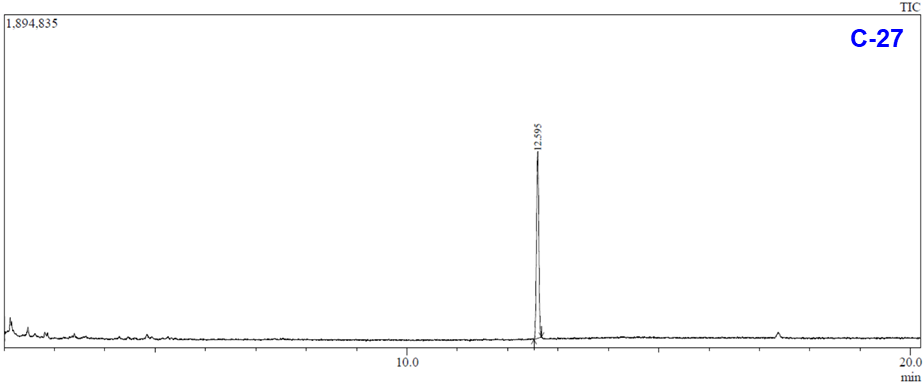
**

**
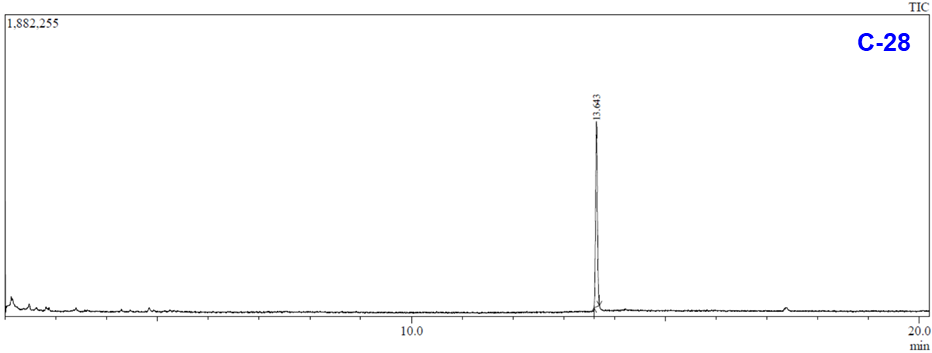
**

**
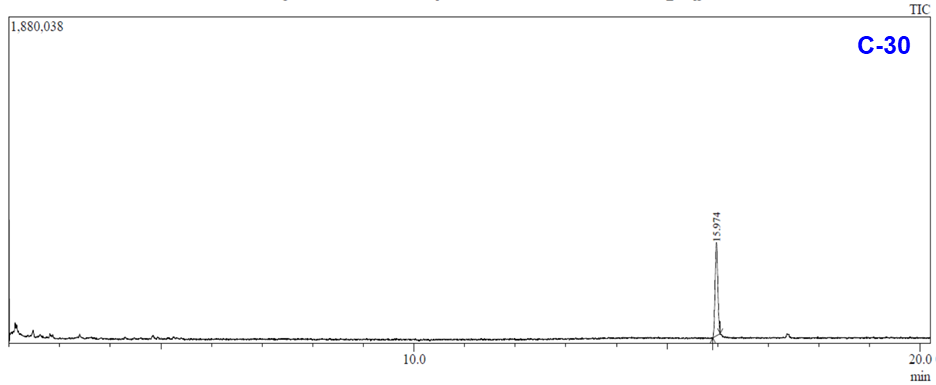
**

**Supplementary Figure 15.** GC–MS total ion chromatograms of individual policosanol.

**
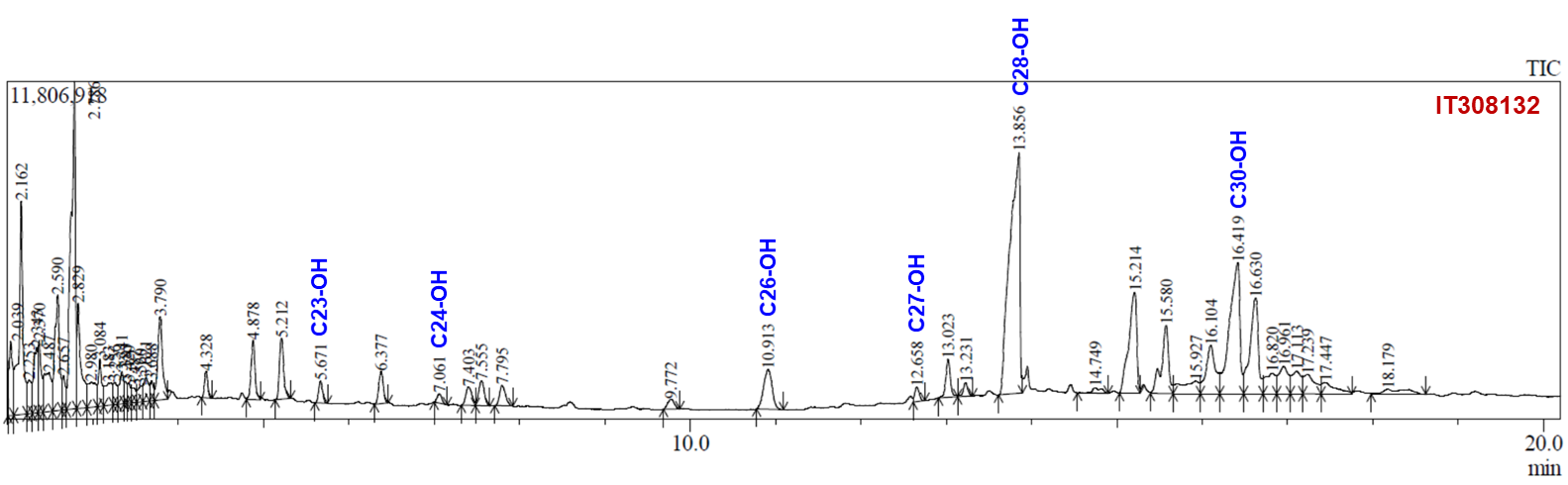
**

**
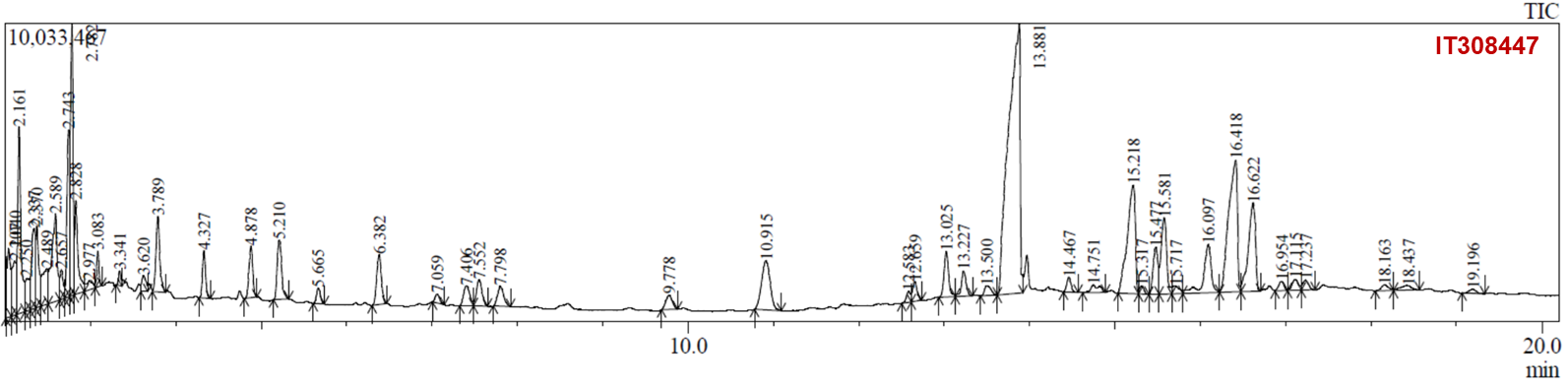
**

**
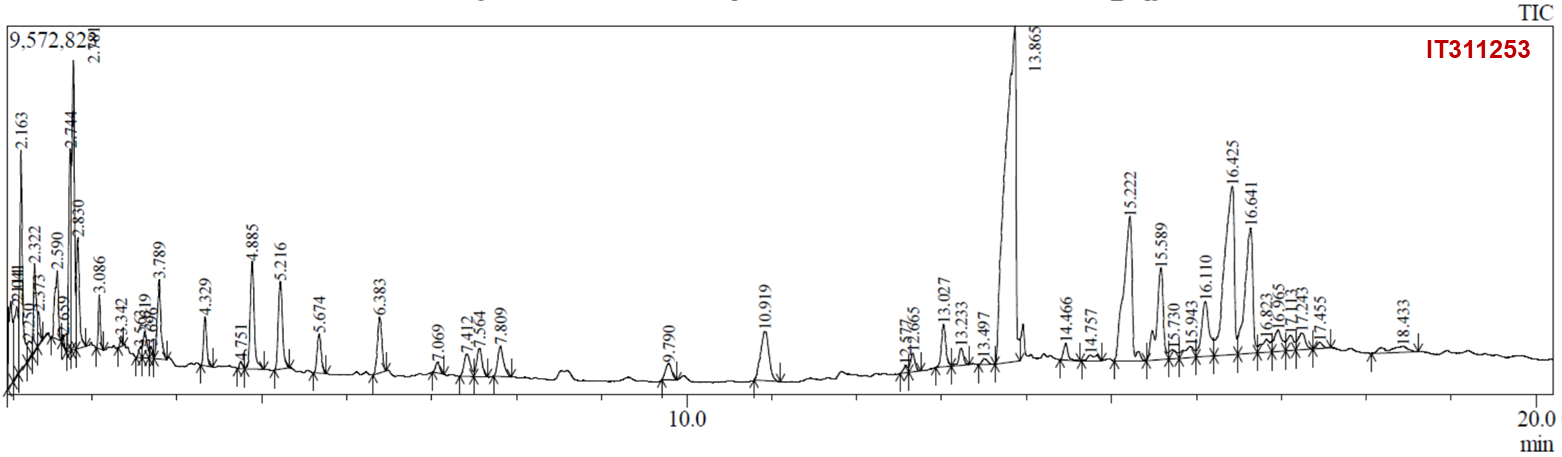
**

**
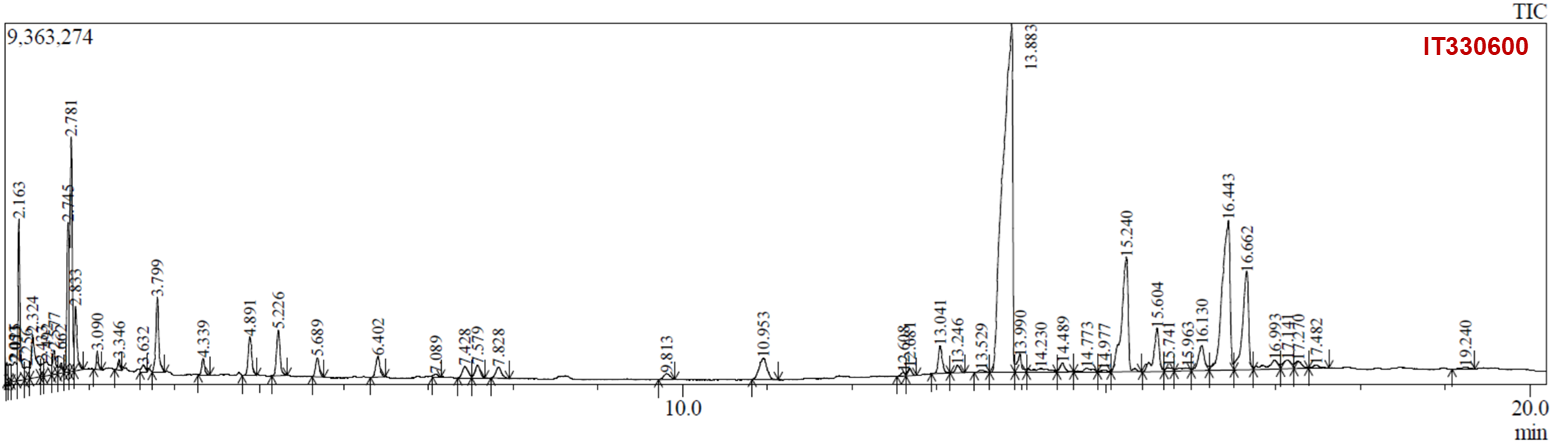
**

**Supplementary Figure 16.** GC–MS total ion chromatograms of policosanols in four Oriental wheat sprout samples,
